# resiLIR HCP: A self-guided digital resilience intervention for healthcare professionals focusing on self-care and self-compassion – A randomized controlled trial

**DOI:** 10.1016/j.invent.2026.101010

**Published:** 2026-10-06

**Authors:** Lisa von Boros, Sarah K. Schäfer, Tabea Werner, Carolina Pank, Friederike Koehler, Federico Zappalà, Benedikt E. Wirth, Tobias Zimmermann, Michael Moos, Lars Gottfriedsen, Alexander Scherrer, Isabella Helmreich, Klaus Lieb, Michèle Wessa, Oliver Tüscher

**Affiliations:** a Leibniz Institute for Resilience Research (LIR), Mainz, Germany; b Department for Clinical Psychology, Psychotherapy and Psychodiagnostics, Technische Universität Braunschweig, Braunschweig, Germany; c Clinical Child and Adolescent Psychology and Psychotherapy, RPTU University Kaiserslautern-Landau, Landau, Germany; d Department of Neuropsychology and Psychological Resilience Research, Central Institute of Mental Health, Mannheim, Germany; e Centre of Excellence in Music, Mind, Body and Brain, Department of Music, Art and Culture Studies, University of Jyväskylä, Jyväskylä, Finland; f Department of Cognitive Assistants, German Research Center for Artificial Intelligence (DKFI), Saarbrücken, Germany; g Department Optimization in Life Sciences, Fraunhofer Institute for Industrial Mathematics (ITWM), Kaiserslautern, Germany; h Mannheim Institute for Intelligent Systems in Medicine (MIISM), Mannheim Medical Faculty, University of Heidelberg, Germany; i Department of Psychiatry and Psychotherapy, University Medical Center of Johannes Gutenberg University, Mainz, Germany; j Division Cancer Survivorship and Psychological Resilience (C160), German Cancer Research Centre (DKFZ), Heidelberg, Germany; k DKFZ-Hector Cancer Institute at the University Medical Center Mannheim, Germany; l Department of Psychiatry, Psychotherapy and Psychosomatic Medicine, University Medicine of the Martin-Luther University Halle-Wittenberg, Halle, Germany; m German Center for Mental Health (DZPG), partner site Halle-Jena-Magdeburg, Halle, Germany

**Keywords:** RCT, Resilience, Digital intervention, Mental health, HCP, Self-care, Self-compassion

## Abstract

**Background:**

Healthcare professionals (HCPs) experience high occupational stress, contributing to mental health problems and workforce attrition. Scalable, low-threshold digital resilience interventions may alleviate this burden.

**Objective:**

This randomized controlled trial evaluated the effectiveness of *resiLIR HCP*, a 6-week, self-guided, web-based intervention focusing on self-care and self-compassion with brief daily exercises, in promoting HCP resilience.

**Methods:**

HCPs (*N* = 234, IG: *n* = 118, CG: *n* = 116) were randomized to the intervention or a waitlist control group. Online assessments were conducted at baseline, mid-intervention, post-intervention, and at 3-, 6-, and 12-month follow-ups. The primary outcome was resilience operationalized as time-varying stressor reactivity. Secondary outcomes included resilience- and work-related outcomes. Intervention effects were primarily evaluated from baseline to post-intervention and 3-month follow-up. Analyses followed per-protocol (PP) and intention-to-treat (ITT) approaches. Preregistration: NCT05812716.

**Results:**

PP analyses (IG: *n* = 45, CG: *n* = 84) revealed significant group × time intervention effects on stressor reactivity at post-intervention, which attenuated at follow-up. Significant effects at post-intervention were observed for self-care, self-compassion, self-efficacy, and psychological distress, whereas effects on depressive and anxiety symptoms were not robust after adjustment for multiple testing. No significant effects were found for work-related outcomes. ITT analyses did not reveal any significant effects, neither for the primary outcome nor secondary outcomes after adjusting for multiple testing. Only 43.2% met the predefined adherence criterion.

**Conclusions:**

PP analyses revealed beneficial effects of *resiLIR HCP*, whereas ITT analyses provided weaker evidence. Limited adherence and non-significant effects on work-related outcomes highlight the need to enhance engagement and integrate digital interventions into multi-level prevention strategies.

## Highlights

• A self-guided digital resilience intervention for healthcare professionals was evaluated.
• Resilience was assessed using the SR Score, a dynamic measure of mental health reactivity to stressor exposure.
• Per-protocol analyses indicated beneficial post-intervention effects which attenuated at follow-up.
• Intention-to-treat analyses provided weaker evidence.
• Improving adherence is key for effective digital resilience interventions.

## 1 Introduction

Healthcare professionals (HCPs) are frequently exposed to high workloads, unsafe working conditions, emotional demands, and time pressure, placing them at increased risk for psychological distress and stress-related diseases (e.g., McCann et al., 2013; Søvold et al., 2021). A recent World Health Organization survey with over 90.000 participants across Europe showed that one in three doctors and nurses report symptoms of depression and anxiety (World Health Organization, 2025). This burden not only undermines individual wellbeing, but may also contribute to reduced work ability, increased medical errors, absenteeism, and workforce attrition (Almeida-Meza et al., 2025). Strengthening resilience of HCPs is therefore essential to safeguard staff well-being and high-quality healthcare (World Health Organization, 2025).

Besides necessary structural changes (e.g., reduced workload) and support services to address the emerging mental health crisis among HCPs (Almeida-Meza et al., 2025; Azzopardi-Muscat et al., 2023), resilience-focused prevention programs represent a promising complementary approach. Over several decades of resilience research, resilience has been conceptualized in three ways: as a trait, process, or outcome (Chmitorz et al., 2018a), with the latter two emphasizing its dynamic nature. From a process perspective, resilience refers to trajectories of psychological adjustment following adversity (Bonanno et al., 2011; Bonanno and Diminich, 2013). As an outcome, resilience is defined as the maintenance or rapid recovery of mental health despite significant stressor exposure (Kalisch et al., 2017).

Research has shown that so-called resilience factors contribute to resilient outcomes by exerting a buffering effect on the extent to which individuals respond to adversity with psychological distress. Higher levels of resilience factors may mitigate the negative impact of stressors and multiple resilience factors can act jointly to promote resilient outcomes (Bonanno et al., 2011). Important resilience factors have been identified on the individual (e.g., internal locus of control), social (e.g., structural work support), and societal level (e.g., environment quality; Schäfer et al., 2024a). Recent theoretical approaches propose higher-order mediating mechanisms that integrate the effects of multiple resilience factors; e.g., positive appraisal style (Kalisch et al., 2015; Petri-Romão et al., 2025) or regulatory flexibility (Bonanno et al., 2024).

Self-care and self-compassion have been identified as promising, complementing target factors to enhance resilience in HCPs, who are trained to care for others, often at the expense of their own needs (Mills and Chapman, 2016; Pank et al., 2025; Skovholt and Trotter-Mathison, 2010). Self-care refers to the *active engagement* in behaviors that support physical, emotional, and social well-being (Harfst et al., 2009), whereas self-compassion reflects a compassionate *attitude* towards oneself, characterized by kindness, mindfulness, and the recognition of shared human suffering (i.e., common humanity; Neff, 2003). Both self-care and self-compassion have been linked to positive mental health outcomes for HCPs, e.g., higher professional quality of life or satisfaction and lower exhaustion and burnout (Babenko et al., 2019; Jung and Jung, 2025), lower perceived stress and higher resilience (Kemper et al., 2015), higher wellbeing and post-traumatic growth (Brillon et al., 2023), and lower depressive and anxiety symptoms (Brillon et al., 2023; Pank et al., 2025). Together, self-care and self-compassion may help restore a balance between self- and other-care (Skovholt and Trotter-Mathison, 2010), and promote an upward spiral towards wellbeing (Posluns and Gall, 2020).

Despite the need for resilience promotion, many HCPs face barriers to accessing mental health support, including demanding work schedules (e.g., shift work), stigma and confidentiality concerns, and limited access to low-threshold services (Zaman et al., 2022). In-person and digital interventions have been developed to promote resilience by addressing resilience factors or mechanisms, showing small to moderate favorable effects on resilience and related mental health outcomes in systematic reviews, including working populations and HCPs (e.g., Schäfer et al., 2024b; Von Boros et al., 2026; Kunzler et al., 2020a, Kunzler et al., 2020b; Vanhove et al., 2016; Liu et al., 2020; Robertson et al., 2016). Therefore, internet-based interventions are a promising solution due to their flexible, location-independent, and scalable delivery (Lehr et al., 2018). For being attractive for HCPs, interventions should be context-specific (Zhang et al., 2025), easily integrable into daily routines, and engaging. Including interactive narrative or quizzes can increase user engagement (Fleming et al., 2017). Even brief online self-compassion interventions can improve mental health in HCPs (Super et al., 2024) though longer programs may produce stronger effects, highlighting the need to balance intensity and feasibility (Kotera and Van Gordon, 2021; Randhawa and Vella-Brodrick, 2025).

### 1.1 Study aim & conceptual framework

Given the potential of self-care and self-compassion to promote resilience in HCPs (e.g., Skovholt and Trotter-Mathison, 2010), we developed and evaluated *resiLIR HCP*, a low-threshold, context-sensitive, self-guided 6-week online intervention integrating psychoeducation with brief daily exercises – so-called weekly/daily training interventions – and optional modules addressing additional resilience factors (e.g., optimism).

This study examines the intervention's effectiveness in enhancing resilience and assesses potential adverse effects. Following methodological recommendations (Chmitorz et al., 2018a; Kalisch et al., 2020), resilience was operationalized as the individual deviation from the normative association between stressor exposure and mental health symptoms. Secondary outcomes included resilience- and health-promoting factors (i.e., mindfulness, self-care, and self-compassion), perceived stress (subjective appraisal of life situations as stressful or overwhelming; Cohen et al., 1983), psychological distress (general mental health problems and reduced well-being; Goldberg, 1972), symptoms of mental disorders (i.e., depressive and anxiety symptoms), and work-related outcomes (i.e., burnout and work-life balance).

### 1.2 Hypotheses

The following preregistered hypotheses were tested:

H1–H11: Compared with the waitlist control group (CG), the intervention group (IG) will show significantly greater improvements from baseline (t0) to post-intervention (t2) and 3-month follow-up (t3), reflected in increases in resilience (measured with SR score (H1; primary outcome) and Brief Resilience Scale (H2)), self-care (H3), self-compassion (H4), mindfulness (H5) and work-life-balance (H11); and reductions in perceived stress (H6), psychological distress (H7), depressive symptoms (H8), anxiety symptoms (H9), and burnout symptoms (H10).
H12: In the IG, resilience will improve by post-intervention (t2) and remain elevated over the follow-up period (t3–t5).
H13–14: State self-compassion (H13) and state self-care (H14) will increase following their respective (i.e., self-compassion focused and self-care-focused) weekly training interventions and the first session of the daily training intervention (comparison of state assessment pre- to post-intervention).
H15: Within the IG, the number of training interventions completed will be associated with increased resilience (SR score), self-compassion, self-care and reduced burnout symptoms at post-intervention (t2).

## 2 Methods

### 2.1 Design

A randomized controlled trial (RCT) compared the *resiLIR HCP* intervention with a waitlist control condition. Six assessments were conducted: baseline (t0), mid-intervention (t1; 4 weeks after baseline), post-intervention (t2; 9 weeks after baseline), and three follow-up assessments (t3, t4, t5; 3, 6 and 12 months after the end of the intervention). The CG was granted access to the intervention after the first follow-up assessment. The trial was preregistered in February 2023 including a statistical analysis plan (Clinicaltrials.gov, 2023b ID: ID: NCT05812716, URL: https://clinicaltrials.gov/study/NCT05812716). All study procedures followed the guidelines of the Declaration of Helsinki (World Medical Association, 2013). The study was approved by the ethics committee of the State Medical Association of Rhineland-Palatinate, Germany (no. 2022-16888) and is reported in accordance with the Consolidated Standards of Reporting Trials (CONSORT) (Hopewell et al., 2025).

### 2.2 Participants & procedure

Participants were recruited via an existing participant pool (Gutenberg Brain Study; https://lir-mainz.de/en/gutenberg-brain-study), posters in clinics and public spaces, online advertisements, and professional associations, networks of HCPs, and academic programs in Germany.

#### 2.2.1 Inclusion criteria

Eligible participants were required to be at least 18 years old, fluent in German, and employed as HCP for a minimum of 10 h per week, with access to PC/laptop/tablet and smartphone. HCPs were defined as formally trained providers of health services (World Health Organization, 2019), e.g., general practitioners. Individuals were excluded if they were experiencing an acute mental health crisis, currently receiving psychiatric or psychotherapeutic treatment, or diagnosed with a neurodegenerative disorder, schizophrenia or another psychotic disorder, bipolar disorder, or post-traumatic stress disorder. Concomitant care during the trial was not systematically assessed. Study participation was conducted remotely, with assessments and the intervention delivered online and no in-person contact between participants and study personnel.

#### 2.2.2 Procedure

An a priori power analysis using G*Power (Faul et al., 2007), based on the preregistered analysis of covariance (ANCOVA) approach, indicated that a total sample size of 146 participants (*n* = 73 per group) was required to detect a medium effect (Hedges' *g* = 0.50) for our primary outcome (SR score; Kalisch et al., 2020) at α = 0.05 with a power of 0.90. Assuming up to 40% dropout, which is common in online interventions (Groot et al., 2023; van Ballegooijen et al., 2014), the recruitment target was increased to *N* = 240 participants. After online registration and baseline assessment, eligible participants were randomized (stratified by age and gender) to the intervention group (IG) or control group (CG) using a computer-generated sequence in Microsoft Excel, generated and implemented by study personnel. The IG received immediate access, whereas the CG received access after the 3-month follow-up; additional follow-ups were conducted at 6 and 12 months. Longer assessments were collected via SoSciSurvey (Leiner, 2024) while state assessments before and after the training interventions were administered within the intervention platform. Blinding of group allocation was not possible due to the study nature. Recruitment and baseline (t0) took place in April 2023, and the final follow-up wave (t5) was completed in July 2024. Participants who endorsed suicidal ideation (i.e., agreement with PHQ-9 item) were contacted by the study psychotherapist via email and offered a one-time consultation on how to proceed. Financial compensation was €5–15 per assessment, depending on length; plus €1 per training intervention for completing the pre- and post-intervention state assessments associated with each weekly or daily training intervention.

#### 2.2.3 Intervention

##### 2.2.3.1 Intervention design and content

The intervention was delivered online via web browser and designed as a six-week program comprising six core modules (one module per week), preceded by an introductory module (Module 0), with up to eight weeks allowed for completion of the intervention. The program was provided in German. Participants were advised to use a PC/computer/tablet for the modules 1 and 2 and a smartphone for modules 3 to 6.

The overall intervention consisted of a theoretical and a practice phase. The first two modules (*theoretical phase*) comprised primarily psychoeducation and self-reflection exercises. The subsequent *practice phase* consisted of four modules focusing on self-compassion and self-care. Each module within the practice phase introduced a new intervention lasting approximately 30 min (further called *weekly training interventions*). Over the following six days, participants were invited with daily email reminders to practice short, related exercises of 5–10 min (further called *daily training interventions*). The reminders are an integral part of the intervention rather than a trial-specific adherence strategy and remained available for continued use of the intervention beyond the evaluation study. Transfer to the HCP context was facilitated through context-specific examples, e.g., illustrating how HCPs coped with work-related stress. An overview of the intervention content is provided in Table 1.

**Table 1:** Content of modules in resiLIR intervention.

Module	Content
Theory modules	
0 – Introduction into the program	• Overview and explanation of the program structure and objectives
1 – Resilience and resilience factors, stressors and resources	• Definition of resilience and resilience factors • Definition of stressors, stress management, and resources • Self-reflection exercise on individual resources
2 – Self-care and self-compassion	• Definition of self-care and self-compassion • Individual goal setting for the intervention • Planning of individual engagement with the intervention, including scheduling specific times for daily intervention exercises
Practice modules	
3 – Self-compassion exercise 1	• Weekly training intervention: Writing a self-compassionate letter to oneself • Daily training interventions: Written reflection on daily adversities, exploration of thoughts, emotions, and behaviors, and reminders to revisit the self-compassionate letter
4 – Self-care exercise 1	• Weekly training intervention: “Living with ease” – reflection on the previous week and identification of situations in which participants could have been less self-critical, made things easier for themselves, or asked for support (adapted from Potreck-Rose, 2020) • Daily training interventions: Reflection on daily situations in which participants could have treated themselves more kindly
5 – Self-compassion exercise 2	• Weekly training intervention: Self-compassionate body scan (audio-guided) • Daily training interventions: Instructions for brief body scan exercises focusing on different body regions (e.g., back, feet)
6 – Self-care exercise 2	• Weekly training intervention: Exploration of pleasant activities and strategies for maintaining a balanced lifestyle; planning of activities for the upcoming week • Daily training interventions: Reminders of planned activities and reflection on their completion and associated experiences
Additional modules (optional)	• Optimism • Acceptance • Purpose in life • Mindfulness

##### 2.2.3.2 Intervention implementation and engagement

The intervention was fully self-guided with modules unlocked sequentially on a weekly basis, and automated email notifications. Participants were free to determine the timing and pacing of module completion once modules became available. Completed modules and exercises could be revisited. Intervention usage was logged automatically (i.e., number of completed modules, completed daily and weekly training interventions, and the time spent on each). Engagement with the intervention was measured in total time spent with the intervention and the number of training interventions completed (i.e., weekly and daily training interventions in the practice phase). The intervention was informed by principles of mindfulness (Kabat-Zinn, 2003), self-compassion (Neff and Germer, 2020), compassion-focused therapy (Gilbert, 2013), and cognitive-behavioral therapy (Beck, 2011).

As the intervention was built upon its sister intervention, *resiLIR basic* Clinicaltrials.gov, 2023a; Zimmermann et al., 2025 for the general population, four optional modules covering mindfulness, optimism, acceptance, and purpose in life were available and could be accessed independently of the core program. Both interventions use the same platform and can be publicly accessed without costs at https://www.resilir.eu/.

To enhance user engagement, gamified elements were included: the program was structured as a mentor-guided city tour with modules located in different buildings and places. Intervention content was presented in an engaging multimedia format (see Fig. 1a, Fig. 1b). Intervention content was selected based on structured evidence reviews about resilience factors (Kunzler et al., 2020a; Kunzler et al., 2020b). No structured public involvement took place during the development, design or evaluation of *resiLIR HCP*; however, feedback on design, content, and structure was obtained from an expert group of associated researchers, some of whom were practicing HCPs.

**Fig. 1a:**
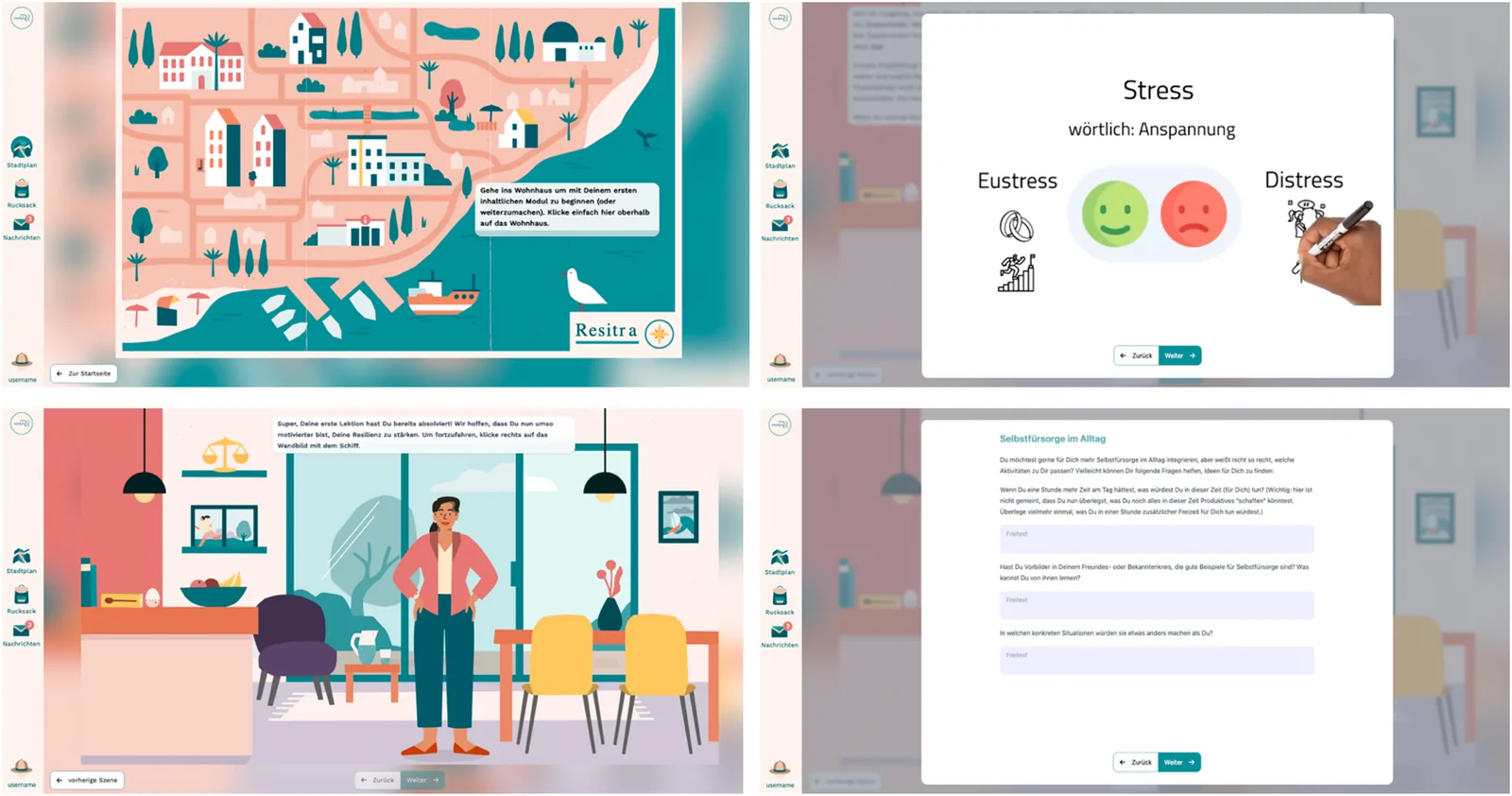
Screenshots from of the *resiLIR HCP* program. *Top left*: City map used to navigate through the virtual city Resitra and select individual training modules. *Top right*: Embedded psychoeducational video on stress within the stress module. *Bottom left*: Scene inside the residential building showing the guide who provides written instructions; intervention content is accessed by clicking on elements in the room. *Bottom right*: Example of interactive content, such as a guided self-reflection exercise focusing on self-care.

**Fig. 1b:**
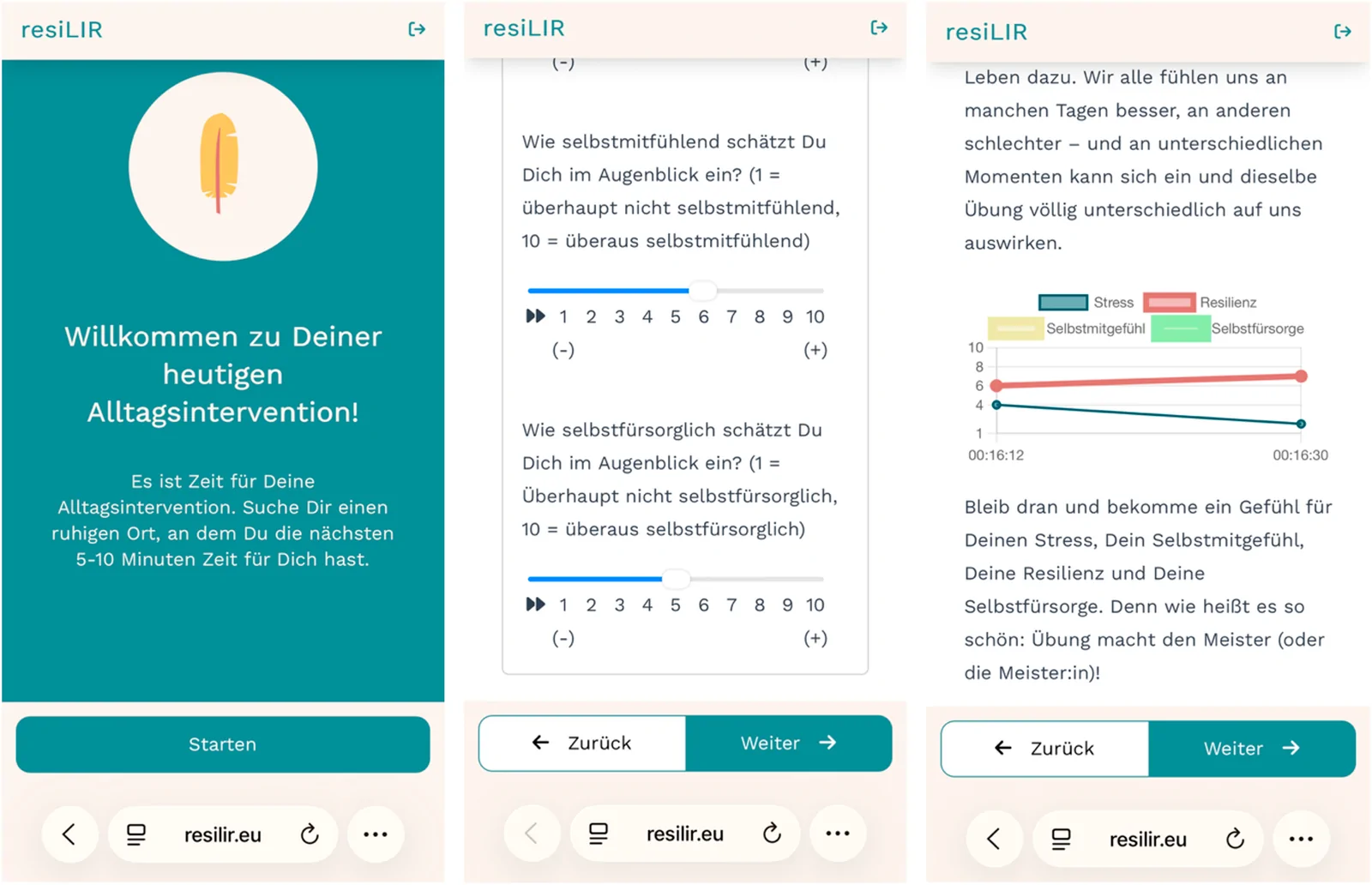
Screenshots of the *resiLIR HCP* program accessed via smartphone during the daily training interventions. *Left*: Welcome screen displayed after following the e-mail reminder for the daily training intervention. *Middle*: Single-item state assessments of self-compassion and self-care administered before and after the daily and weekly training interventions. *Right*: Graphic providing participants with immediate feedback by displaying pre–post comparisons of their state assessment ratings.

### 2.3 Measures

#### 2.3.1 Primary outcome: resilience measured with the SR score

The primary outcome was the stressor reactivity (SR) score (Kalisch et al., 2020; Petri-Romão et al., 2025), an index of resilience defined as lower-than-expected mental health symptoms given an individual's level of stressor exposure. The SR score was calculated as the residual from a regression of mental health symptoms (composite of anxiety symptoms (Generalized Anxiety Disorder Questionnaire GAD-7; Spitzer et al., 2006), and depressive symptoms (Patient Health Questionnaire PHQ-9; Löwe et al., 2002)), on stressor exposure (Mainz Inventory of Micro Stressors MIMIS; Chmitorz et al., 2020), reflecting normative stress reactivity in this sample. Negative residuals indicate more favorable outcomes (lower reactivity to stressors, higher resilience), positive residuals vice versa. Importantly, the SR score is conceptualized as a time-varying indicator of intra-individual stressor reactivity; accordingly, repeatedly lower SR scores across assessment waves are considered particularly meaningful, indicating sustained changes (Kalisch et al., 2021). The SR score was the primary outcome, with post-intervention (t2) and the 3-month follow-up (t3) defined as the primary time points of interest. To avoid confounding intervention effects, the normative regression line was estimated using baseline data only; to enhance robustness of the data, the estimation was supplemented by baseline data from two sister trials within the *resiLIR* project (a general population sample, *n* = 236, and a student sample, *n* = 215).

#### 2.3.2 Secondary outcomes

Secondary outcomes comprised self-reported resilience (Brief Resilience Scale BRS; Chmitorz et al., 2018b), self-compassion (Self-Compassion Scale SCS; Hupfeld and Ruffieux, 2011), self-care (Hamburg Self-Care Survey HSF; Harfst et al., 2009; subscales: positive experience, pacing), mindfulness (Mindful Awareness and Attention Scale-Short Form MAAS; Höfling et al., 2011), perceived stress (Perceived Stress Scale PSS 2&2; Schäfer et al., 2023; subscales: helplessness, self-efficacy), psychological distress (General Health Questionnaire GHQ-12; Schrnitz et al., 1999), anxiety symptoms (GAD-7; Spitzer et al., 2006), depressive symptoms (PHQ-9; Löwe et al., 2002), work-life balance (Trier Short Scale for the Assessment of Work-Life Balance TKS-WLB; Syrek et al., 2011), and burnout (Maslach Burnout Inventory MBI; Büssing and Perrar, 1992; subscales: emotional exhaustion, depersonalization, personal accomplishment). All measures showed acceptable to excellent internal consistencies (*α* = 0.83–0.94, *ω* = 0.86–0.95, Spearman-Brown-Coefficient (recommended for 2-item scales; Eisinga et al., 2013) = 0.67–0.75).

Momentary self-compassion and self-care were assessed using two single-item visual analogue scales administered immediately before and after the weekly and daily training interventions, see details regarding measures in SM 3.

#### 2.3.3 Additional measures (for moderator tests)

Additional constructs were assessed as potential moderators of intervention effects, i.e., optimism (SOP-2; Kemper et al., 2014); positive reappraisal (PASS; Petri-Romão et al., 2024); internal control beliefs (IE-4; Kovaleva et al., 2014); satisfaction with the intervention (CSQ-I; Boß et al., 2016); and attitudes towards psychological online interventions (APOI; Schröder et al., 2015). All measures are established instruments and showed acceptable to excellent internal consistencies (*α* = 0.78–0.97, *ω* = 0.84–0.98, Spearman-Brown-Coefficient = 0.64–0.84).

#### 2.3.4 Feedback on the intervention and assessment of potential harms

Participants rated their satisfaction with the structure of the intervention, intervention content, usability, and design of the program, on a 5-point Likert scale ranging from 1 (*not at all satisfied*) to 5 (*completely satisfied*). Moreover, two items assessed whether technical difficulties had impaired the user experience. To assess potential harms through the intervention, the Inventory for the Balanced Assessment of Negative Effects of Online Interventions (INEP-ON; Ladwig et al., 2014) was administered at post-intervention. It assesses the occurrence, perceived attribution, and severity of negative effects.

### 2.4 Statistical analyses

The statistical analyses adhered to our pre-registration (Clinicaltrials.gov, 2023b). However, linear mixed-effects models were used instead of the preregistered ANCOVA approach to better account for the longitudinal data structure and repeated observations within participants (see SM 2 for deviations from preregistration). Our analyses were conducted in accordance with established recommendations for the analysis of RCTs (Altman, 1996; Harrer et al., 2023) and conducted in R (R Core Team, 2023) using the packages lme4 (Bates et al., 2015), lmerTest (Kuznetsova et al., 2017), mice (Buuren and Groothuis-Oudshoorn, 2011), robustbase (Maechler et al., 2025), and emmeans (Lenth and Piaskowski, 2017).

Analyses employed both per-protocol (PP) and intention-to-treat (ITT) approaches. For PP analyses, participants with at least one assessment after baseline and, in the intervention group, sufficient intervention engagement (completion of minimum 10 training interventions out of possible 28) were included. A stricter adherence criterion (≥ 14 training interventions), originally preregistered, seemed overly restrictive considering actual adherence and was therefore applied only in a sensitivity analysis (see SM 10). For ITT analyses, all data of initially randomized participants were used (i.e., no exclusion criterion for intervention usage was applied) and missing data were handled employing multiple imputations using both multilevel predictive mean matching (2l.pmm) to account for hierarchical data structures, where applicable, and standard predictive mean matching (pmm). Twenty imputed datasets were generated, and estimates were pooled according to Rubin's rule (Harrer et al., 2023). Prior to all analyses, data quality checks were applied. Questionnaire assessments were excluded at the respective time point if completion times indicated critically fast responding (TIME_RSI > 2; SoSciSurvey, 2026); participants who indicated unusable data via a check item were excluded from all analyses.

#### 2.4.1 Statistical analysis of H1 to H15

Intervention effects for H1–H11 were analyzed using a repeated measures analysis with baseline adjustment following Twisk (2021). For each outcome, two models were estimated. First, a linear mixed model (LMM) including all assessments (baseline, post-intervention and first follow-up) was fitted to estimate the overall intervention effect. Models included time (coded 0 for baseline and 1 for all following assessments) as a fixed effect, the interaction between time and group (coded 0 for CG and 1 for IG) and a random intercept for participants to account for repeated measurements. The main effect of group was not included in the model; therefore, constraining baseline means to be equal across groups for baseline adjustment. The time × group regression coefficient here indicates the overall intervention effect, namely the difference between IG and CG on average over time. Second, time-specific intervention effects were examined by introducing dummy variables for post-intervention (week 9) and first follow-up (week 21), yielding baseline-adjusted estimates of group differences at each time point. The group × time dummy coefficients indicate intervention effects at the respective time points.

To test whether resilience improved during the year after intervention (H12), changes in the SR score within the intervention group were analyzed using an LMM with time as a fixed effect and a random intercept for participants. All available measurement occasions from mid-intervention through one-year post-intervention were included to model change and stability over time.

Separate LMMs examining pre–post changes in state self-compassion and state self-care were tested for daily and weekly intervention exercises (to test H13–H14) with time (pre vs. post) as a fixed effect, and random intercepts for participants to account for repeated observations.

Dose–response relationships (H15) were examined using robust regression models predicting post-intervention outcomes from baseline scores and number of completed interventions (dose), to reduce sensitivity to influential observations. Effect of interest was the main effect of intervention dose, indicating whether higher intervention engagement was associated with post-intervention outcomes while controlling for baseline level. Exploratory analyses tested potential non-linear (logarithmic, quadratic, segmented) dose–response patterns. Analyses included all intervention-group participants who logged into the platform at least once and completed the post-intervention assessment.

#### 2.4.2 Moderation and dropout analyses

Exploratory moderation analyses were conducted using LMMs with random intercepts for participants. Between-group moderation was tested via time × group × moderator interactions, examining these potential moderators: attitudes towards online interventions, confidence in online interventions (subscale of APOI), resilience factors and mechanisms at baseline (i.e., optimism, locus of control, and positive appraisal style), age, and gender. Additional LMMs examined moderators assessed only among participants in the IG (i.e., satisfaction with the intervention (CSQ-I), feedback on the intervention, technical difficulties), with time × moderator interactions being of interest. All moderators were analyzed in separate models. Moderation was tested for the outcomes resilience (SR score), self-compassion, self-care (subscales pacing and positive experience), and psychological distress.

Selective attrition was examined by comparing completers and dropouts (i.e., participants without post-intervention data) on baseline characteristics using Student's *t*-tests.

#### 2.4.3 Robustness of results

To address multiple testing, *p*-values for secondary outcomes (H2−H11) were adjusted using the Hommel procedure (Hommel, 1988) which controls the family-wise type I error rate while retaining greater statistical power than Bonferroni-based adjustments (Vickerstaff et al., 2019). Multiplicity adjustment was not applied to time-specific contrasts in H2–H11 as we considered these a decomposition of the overall effects, or to hypotheses H12–H15, as these analyses addressed distinct research questions. The primary outcome (H1) was tested at *α* = 0.05.

## 3 Results

### 3.1 Sample characteristics and intervention usage

A total of 234 participants (82.5% self-identified women), aged 21–67 years (*M* = 41.35, *SD* = 12.11) were randomly allocated to the intervention (*n* = 118) and waitlist control (*n* = 116) conditions. Baseline characteristics are summarized in Table 2 and a participant flow chart is depicted in Fig. 2. Satisfying adherence to the intervention was available for *n* = 51 individuals from the intervention group. However, not all of them provided data at the relevant assessments or passed quality checks for the data (i.e., TIME_RSI); consequently, the PP analyses were conducted on a reduced sample size of 45 participants.

**Table 2:** Sample characteristics at baseline (all randomized participants).

	All (*N* = 234)	Control (*n* = 116)	Intervention (*n* = 118)
Age: *M* (*SD*)	41.35 (12.11)	41.69 (12.10)	41.00 (12.16)
Sex: # (%)			
Women	193 (82.5%)	96 (82.8%)	97 (82.2%)
Men	41 (17.5%)	20 (17.2%)	21 (17.8%)
Education (attended years of school): # (%)			
Up to 9 years	4 (1.7%)	3 (2.6%)	1 (0.9%)
10–11 years	51 (21.8%)	28 (24.1%)	23 (19.5%)
12+ years	179 (76.5%)	85 (73.3%)	94 (79.7%)
Profession: # (%)			
Physician	39 (16.7%)	13 (11.2%)	26 (22.0%)
Nurse	40 (17.1%)	22 (18.9%)	18 (15.3%)
Physical therapist	34 (14.5%)	16 (13.8%)	18 (15.3%)
Speech therapist	42 (17.9%)	22 (18.9%)	20 (16.9%)
Psychotherapist	23 (9.8%)	13 (11.2%)	10 (8.5%)
Dentist	3 (1.3%)	1 (0.9%)	2 (1.7%)
Medical technical or laboratory assistant	1 (0.4%)	1 (0.9%)	–
Geriatric nurse	2 (0.9%)	2 (1.7%)	–
Other	50 (21.4%)	26 (22.4%)	24 (20.3%)

*Note.* M = mean, SD = standard deviation, # = number.

**Fig. 2:**
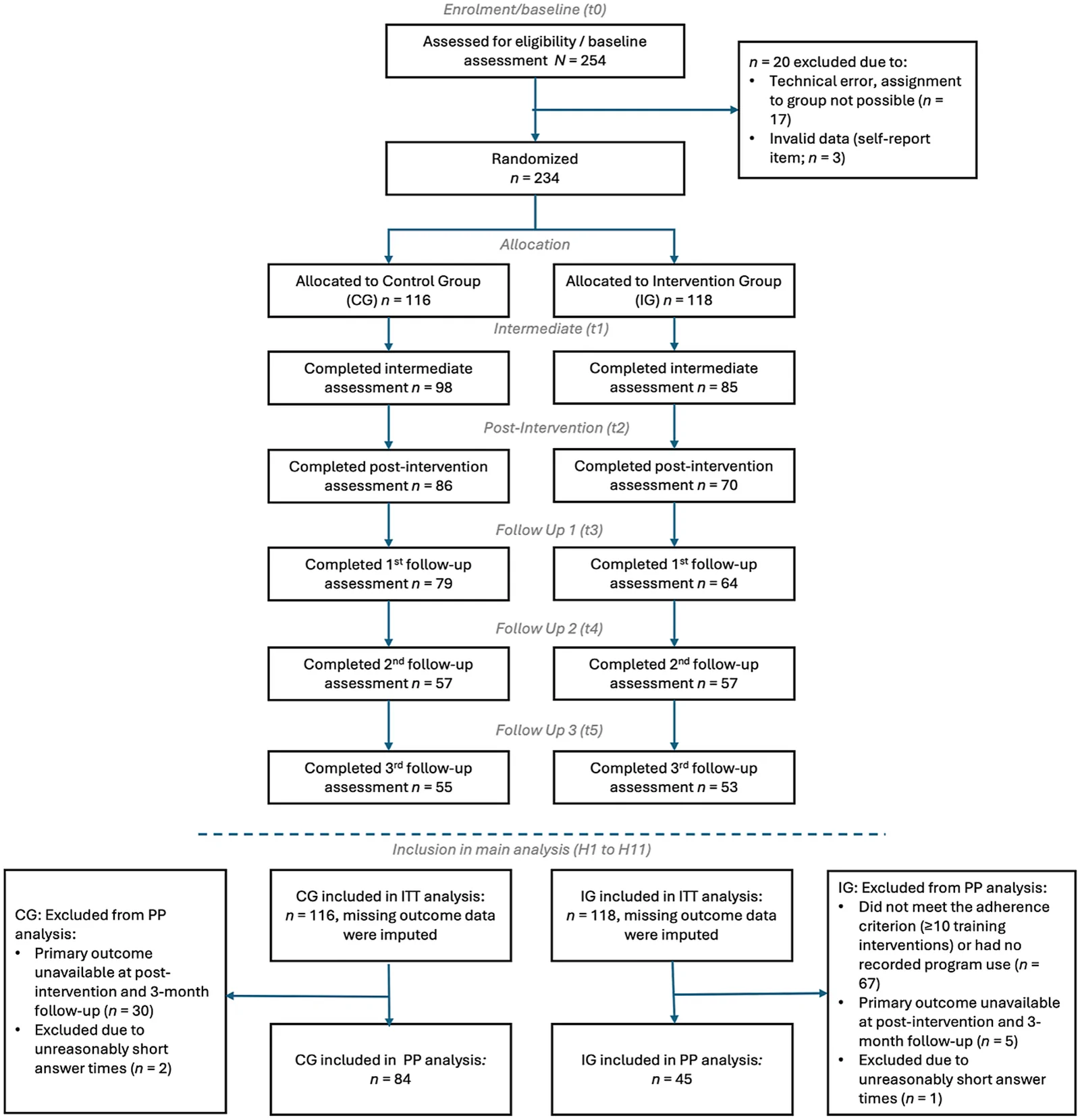
Participants' flow through the study, including group allocation, and assessment completion rates across all time points. Participants were randomized into IG and CG after baseline completion. *Note.* Inclusion in the main analysis refers to participants included in the analyses for H1–H11. Sample sizes may differ for other analyses due to hypothesis-specific inclusion criteria and assessment time points (e.g., H12 included additional follow-up assessments).

Baseline characteristics for the PP sample are displayed in SM 4. Baseline characteristics did not indicate significant baseline differences between completers and dropouts (see Section 3.2.5. Dropout analyses). Overall adherence to the intervention (i.e., the extent to which participants completed the intervention as intended) was limited: 30.5% of participants randomized to the intervention group did not access the intervention or engage with any content, whereas only 43.2% met the predefined criterion for sufficient adherence by completing at least 10 training interventions. Detailed information on intervention usage, adherence, and descriptive statistics of the PP sample is provided in SM 5 and SM 6.

### 3.2 Intervention effects (H1 to H15)

#### 3.2.1 H1 to H11: main effect of the intervention

For the primary outcome, PP analyses revealed a significant group × time overall interaction favoring the intervention group for resilience measured with the SR score (H1; *b* = −0.28, 95% CI [−0.49, −0.06], *p* = .011), with the effect being significant at post-intervention (*b* = −0.34, 95% CI [−0.60, −0.08], *p* = .010) but not maintained at follow-up (*b* = −0.19, 95% CI [−0.47, 0.08], *p* = .172). In contrast, the corresponding ITT analysis did not indicate a significant overall group × time interaction (*b* = −0.10, 95% CI [−0.28, 0.08], *p* = .261), and the time-specific effects were also not significant. Thus, H1 was supported in the PP but not in the ITT analysis.

For secondary outcomes, significant effects were found in the PP analyses for both self-care subscales positive experience and pacing (H3a, H3b), self-compassion (H4), self-efficacy (PSS subscale; H6b), psychological distress (H7), depressive symptoms (H8), and anxiety symptoms (H9), with strongest effects at post-intervention and partially sustained at follow-up. No significant group × time effects emerged for resilience (measured with the BRS; H2), mindfulness (H5), helplessness (PSS subscale, H6a), emotional exhaustion (H10a), depersonalization (H10b), personal accomplishment (H10c), and work–life balance (H11). In the ITT analyses, only two effects were significant, namely for self-care (subscale pacing; H3b) and psychological distress (H7).

Applying Hommel adjustment for multiple testing across secondary outcomes (H2-H11) in the PP analyses indicated that effects for self-care (subscale pacing), self-compassion, perceived stress (subscale self-efficacy), and psychological distress remained statistically significant, whereas effects for self-care (subscale positive experience), depressive symptoms and anxiety symptoms did not meet the adjusted significance threshold. For ITT analyses, none of the secondary outcome effects remained statistically significant after Hommel adjustments. Detailed results are presented in Table 3, Table 4 and Fig. 3.

**Table 3:** Results of per protocol (PP) and intention-to-treat (ITT) analysis of hypotheses H1 to H11: Overall group × time effects.

Hypothesis: outcome	PP analysis (*n* = 129)							ITT analysis (*n* = 234)						
	*b*	*SE*	95% CI	*df*	*t*	*p* (raw)	*p* (Hommel)	*b*	*SE*	95% CI	*df*	*t*	*p* (raw)	*p* (Hommel)
H1: Resilience (SR score)	−0.29	0.11	[−0.51, −0.07]	361	−2.63	**0.009**	–	−0.10	0.09	[−0.28, 0.08]	188	−1.13	0.261	–
H2: Resilience (BRS)	0.16	0.08	[−0.00, 0.32]	322	1.94	0.053	0.321	0.01	0.07	[−0.12, 0.14]	231	0.13	0.899	0.977
H3a: Self-care – positive experience	0.23	0.10	[0.04, 0.42]	353	2.39	**0.017**	0.137	0.05	0.08	[−0.10, 0.21]	208	0.66	0.509	0.977
H3b: Self-care – pacing	0.54	0.11	[0.34, 0.75]	354	5.10	**<0.001**	<0.001	0.22	0.08	[0.06, 0.38]	246	2.67	**0.008**	0.112
H4: Self-compassion	0.24	0.06	[0.12, 0.36]	297	3.84	**<0.001**	0.002	0.08	0.05	[−0.02, 0.17]	279	1.56	0.120	0.960
H5: Mindfulness	0.11	0.10	[−0.08, 0.30]	330	1.10	0.270	0.596	−0.01	0.12	[−0.25, 0.22]	152	−0.12	0.907	0.977
H6a: Perceived stress – helplessness	−0.09	0.11	[−0.31, 0.12]	355	−0.85	0.398	0.795	−0.06	0.11	[−0.27, 0.15]	210	−0.60	0.550	0.977
H6b: Perceived stress – self-efficacy	0.30	0.09	[0.13, 0.47]	356	3.49	**<0.001**	0.006	0.00	0.11	[−0.21, 0.21]	137	−0.03	0.977	0.977
H7: Psychological distress	−3.48	0.71	[−4.88, −2.08]	360	−4.88	**<0.001**	<0.001	−1.41	0.59	[−2.57, −0.25]	202	−2.40	**0.017**	0.221
H8: Depressive symptoms	−1.28	0.51	[−2.28, −0.28]	348	−2.52	**0.012**	0.098	−0.56	0.38	[−1.32, 0.19]	348	−1.47	0.143	0.977
H9: Anxiety symptoms	−1.23	0.47	[−2.15, −0.31]	360	−2.63	**0.009**	0.077	−0.50	0.39	[−1.28, 0.28]	174	−1.27	0.206	0.977
H10a: Burnout – emotional exhaustion	−0.14	0.12	[−0.37, 0.10]	294	−1.12	0.263	0.596	−0.13	0.10	[−0.32, 0.06]	221	−1.31	0.191	0.977
H10b: Burnout – depersonalisation	−0.16	0.15	[−0.45, 0.12]	325	−1.12	0.265	0.596	−0.18	0.11	[−0.39, 0.04]	197	−1.60	0.112	0.927
H10c: Burnout – personal accomplishment	0.16	0.09	[−0.02, 0.34]	331	1.73	0.085	0.427	0.03	0.08	[−0.13, 0.18]	175	0.32	0.749	0.977
H11: Work-life balance	0.03	0.11	[−0.20, 0.25]	335	0.24	0.811	0.811	−0.04	0.09	[−0.22, 0.13]	185	−0.48	0.634	0.977

*Note.* PP = per-protocol; ITT = intention-to-treat. PP analyses included intervention-group participants who completed at least 10 training sessions; participants in both groups were additionally required to provide at least one post-baseline assessment of the primary outcome. ITT analyses included all randomized participants and handled missing data using multiple imputation. Negative coefficients (*b*) indicate improvements (reductions) for negatively scored outcomes (i.e., resilience (SR score), helplessness, psychological distress, depressive symptoms, anxiety symptoms, emotional exhaustion, and depersonalisation). Positive coefficients indicate improvement for positively scored outcomes (i.e., resilience (BRS), self-care, self-compassion, mindfulness, self-efficacy, personal accomplishment, and work-life balance). *p* (raw) = unadjusted *p*-values, *p* (Hommel) = Hommel-adjusted *p*-values (to account for multiple testing for secondary outcomes H2–H11). Unadjusted *p*-values < .05 are shown in **bold**.

**Table 4:** Results of per protocol (PP) and intention-to-treat (ITT) analysis for hypotheses H1 to H11: Specific group × time effects for post-intervention and first follow-up (3 months after completion of intervention).

Hypothesis: outcome	PP analysis (*n* = 129)							ITT analysis (*n* = 234)						
	Week	*b*	*SE*	95% CI	*df*	*t*	*p*	Week	*b*	*SE*	95% CI	*df*	*t*	*p*
H1: Resilience (SR score)	9	−0.34	0.13	[−0.60, −0.08]	339	−2.55	**0.011**	9	−0.16	0.12	[−0.39, 0.07]	137	−1.41	0.160
	21	−0.22	0.14	[−0.51, 0.06]	333	−1.55	0.121	21	−0.04	0.11	[−0.26, 0.18]	191	−0.39	0.701
H2: Resilience (BRS)	9	0.14	0.10	[−0.05, 0.33]	297	1.45	0.148	9	−0.01	0.08	[−0.17, 0.15]	185	−0.09	0.925
	21	0.18	0.10	[−0.02, 0.38]	292	1.74	0.083	21	0.02	0.08	[−0.13, 0.18]	224	0.31	0.756
H3a: Self-care – positive experience	9	0.24	0.12	[0.01, 0.46]	325	2.04	**0.043**	9	0.06	0.09	[−0.12, 0.24]	319	0.69	0.489
	21	0.23	0.13	[−0.01, 0.48]	319	1.84	0.067	21	0.04	0.10	[−0.15, 0.24]	162	0.42	0.673
H3b: Self-care – pacing	9	0.66	0.13	[0.41, 0.90]	324	5.24	**<0.001**	9	0.32	0.10	[0.12, 0.51]	310	3.22	**0.001**
	21	0.42	0.14	[0.16, 0.69]	318	3.13	**0.002**	21	0.13	0.11	[−0.08, 0.34]	152	1.20	0.232
H4: Self-compassion	9	0.16	0.07	[0.02, 0.30]	278	2.30	**0.022**	9	0.05	0.06	[−0.06, 0.16]	297	0.88	0.379
	21	0.33	0.08	[0.18, 0.48]	275	4.36	**<0.001**	21	0.10	0.06	[−0.01, 0.21]	236	1.74	0.083
H5: Mindfulness	9	0.02	0.11	[−0.20, 0.24]	302	0.17	0.865	9	−0.06	0.14	[−0.34, 0.23]	139	−0.40	0.689
	21	0.23	0.12	[−0.00, 0.47]	297	1.94	0.053	21	0.03	0.14	[−0.25, 0.31]	142	0.21	0.835
H6a: Perceived stress – helplessness	9	−0.22	0.13	[−0.48, 0.05]	349	−1.62	0.106	9	−0.15	0.13	[−0.41, 0.10]	273	−1.17	0.245
	21	0.06	0.15	[−0.23, 0.34]	345	0.38	0.703	21	0.02	0.14	[−0.25, 0.30]	147	0.17	0.863
H6b: Perceived stress – self-efficacy	9	0.38	0.10	[0.18, 0.58]	328	3.75	**<0.001**	9	0.00	0.13	[−0.25, 0.25]	202	0.01	0.990
	21	0.19	0.11	[−0.02, 0.41]	322	1.74	0.083	21	−0.01	0.13	[−0.27, 0.25]	141	−0.06	0.952
H7: Psychological distress	9	−3.87	0.87	[−5.57, −2.17]	345	−4.46	**<0.001**	9	−1.88	0.75	[−3.36, −0.40]	156	−2.51	**0.013**
	21	−3.00	0.94	[−4.85, −1.16]	340	−3.20	**0.002**	21	−0.95	0.77	[−2.47, 0.58]	128	−1.23	0.221
H8: Depressive symptoms	9	−1.42	0.60	[−2.60, −0.24]	319	−2.36	**0.019**	9	−0.86	0.45	[−1.74, 0.03]	438	−1.90	0.058
	21	−1.13	0.65	[−2.40, 0.15]	313	−1.73	0.084	21	−0.27	0.51	[−1.27, 0.74]	146	−0.53	0.598
H9: Anxiety symptoms	9	−1.40	0.56	[−2.50, −0.30]	335	−2.50	**0.013**	9	−0.69	0.47	[−1.62, 0.23]	200	−1.48	0.140
	21	−1.02	0.61	[−2.21, 0.17]	329	−1.68	0.094	21	−0.31	0.51	[−1.32, 0.71]	107	−0.60	0.552
H10a: Burnout – emotional exhaustion	9	−0.05	0.14	[−0.32, 0.22]	276	−0.36	0.719	9	−0.07	0.12	[−0.30, 0.16]	194	−0.62	0.537
	21	−0.24	0.15	[−0.54, 0.05]	273	−1.63	0.103	21	−0.18	0.12	[−0.41, 0.05]	162	−1.54	0.125
H10b: Burnout – depersonalisation	9	−0.06	0.17	[−0.40, 0.28]	298	−0.35	0.727	9	−0.17	0.13	[−0.42, 0.08]	292	−1.34	0.180
	21	−0.30	0.19	[−0.66, 0.07]	293	−1.60	0.110	21	−0.18	0.14	[−0.47, 0.10]	107	−1.27	0.205
H10c: Burnout – personal accomplishment	9	0.14	0.11	[−0.08, 0.35]	305	1.27	0.206	9	0.03	0.10	[−0.16, 0.21]	195	0.26	0.792
	21	0.19	0.12	[−0.04, 0.42]	299	1.61	0.108	21	0.03	0.10	[−0.17, 0.22]	131	0.26	0.794
H11: Work-life balance	9	−0.08	0.13	[−0.34, 0.19]	307	−0.57	0.572	9	−0.09	0.11	[−0.30, 0.12]	182	−0.85	0.397
	21	0.16	0.14	[−0.12, 0.44]	301	1.09	0.276	21	0.01	0.11	[−0.20, 0.21]	175	0.05	0.960

*Note.* PP = per-protocol; ITT = intention-to-treat. PP analyses included intervention-group participants who completed at least 10 training sessions; participants in both groups were additionally required to provide at least one post-baseline assessment of the primary outcome. ITT analyses included all randomized participants and handled missing data using multiple imputation. Week 9 = post-intervention; week 21 = follow-up (3 months after completion of the intervention). Negative coefficients (*b*) indicate improvements (reductions) for negatively scored outcomes (i.e., resilience (SR score), helplessness, psychological distress, depression, anxiety, emotional exhaustion, and depersonalisation). Positive coefficients indicate improvement for positively scored outcomes (i.e., resilience (BRS), self-care, self-compassion, mindfulness, self-efficacy, personal accomplishment, and work-life balance). Unadjusted *p*-values < .05 are shown in **bold**.

**Fig. 3:**
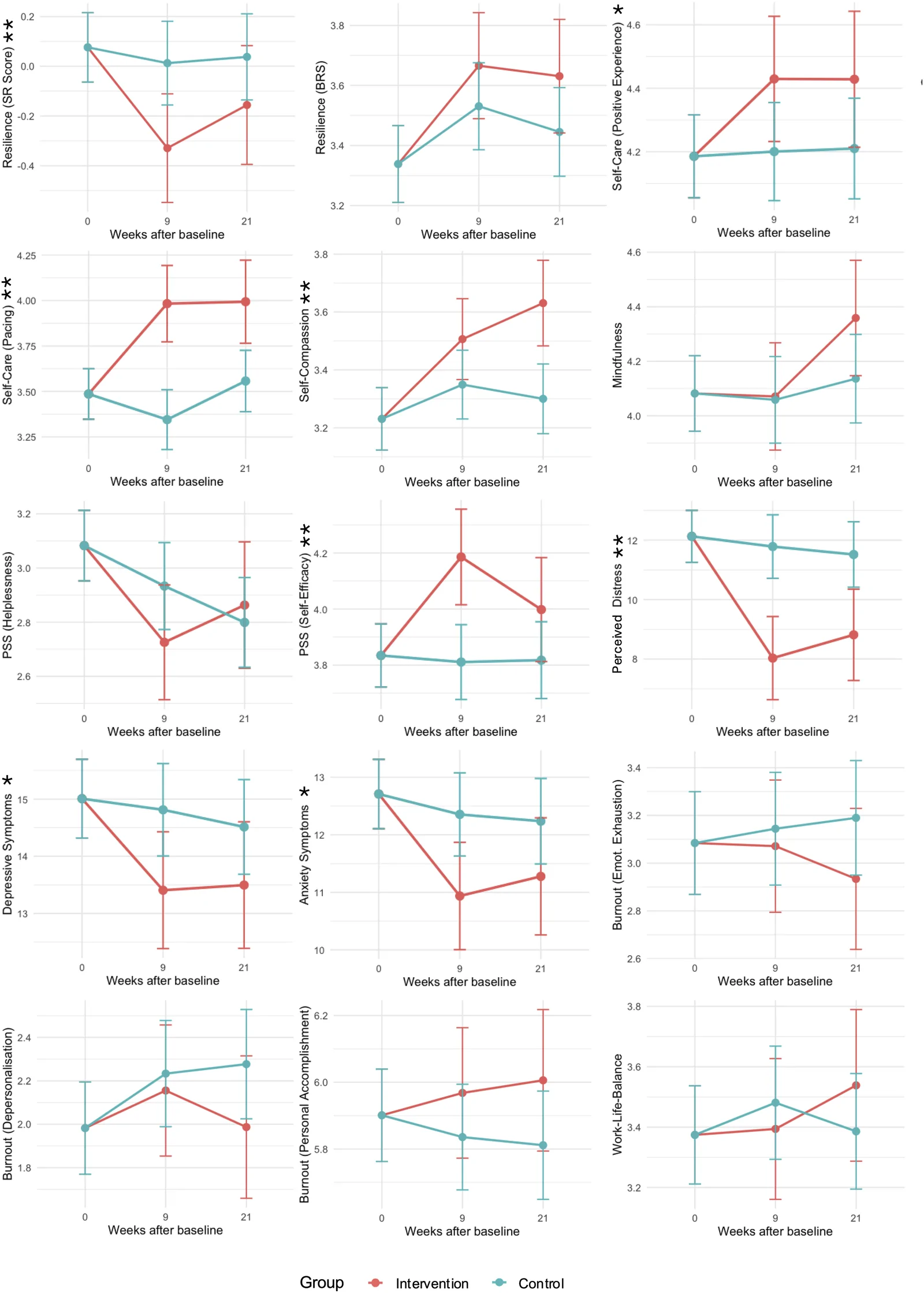
Plots of H1 to H11 (per protocol analyses). *Note.* Values represent model-based estimated marginal means (EMMs) from a LMM adjusted for baseline. Error bars show 95% confidence intervals. Significant overall group × time interactions (according to raw *p*-values, not Hommel-adjusted *p*-values) are indicated by asterisks in the panels (*: *p* < .05, **: *p* < .01). The corresponding plots for the ITT analysis are presented in SM 7.

#### 3.2.2 H12: stability of effect on resilience

For the SR score in the intervention group (H12), LMMs in the PP analyses showed significant reductions already at t1, but the largest effect at the end of the intervention (t2; *b* = −0.47, 95% CI [−0.68, −0.25], *p* < .001). Smaller but significant effects were observed at t3, whereas effects at t4 and t5 were no longer significant. In contrast, none of the changes from baseline were statistically significant in the ITT analysis (all *p*s ≥ .074), see details in Table 5 and Fig. 4.

**Table 5:** Linear mixed model results for SR score (only intervention group, both PP and ITT analyses).

Assessment (weeks after baseline)	PP analysis (*n* = 48)			ITT analysis (*n* = 118)		
	*b*	95% CI	*p*	*b*	95% CI	*p*
Mid-intervention (4)	−0.24	[−0.45, −0.02]	**0.030**	−0.14	[−0.37, 0.09]	0.233
Post-intervention (9)	−0.47	[−0.68, −0.25]	**<0.001**	−0.23	[−0.47, 0.02]	0.074
3-months follow-up (21)	−0.30	[−0.54, −0.07]	**0.011**	−0.12	[−0.37, 0.14]	0.366
6-months follow-up (33)	−0.21	[−0.44, 0.02]	0.076	−0.04	[−0.29, 0.21]	0.735
1-year follow-up (59)	−0.16	[−0.40, 0.09]	0.215	−0.12	[−0.36, 0.12]	0.334

Note. PP = per-protocol, ITT = intention-to-treat. Baseline served as the reference assessment, negative b coefficients indicate a decrease in SR Score relative to baseline. p-values < .05 are shown in **bold**.

**Fig. 4:**
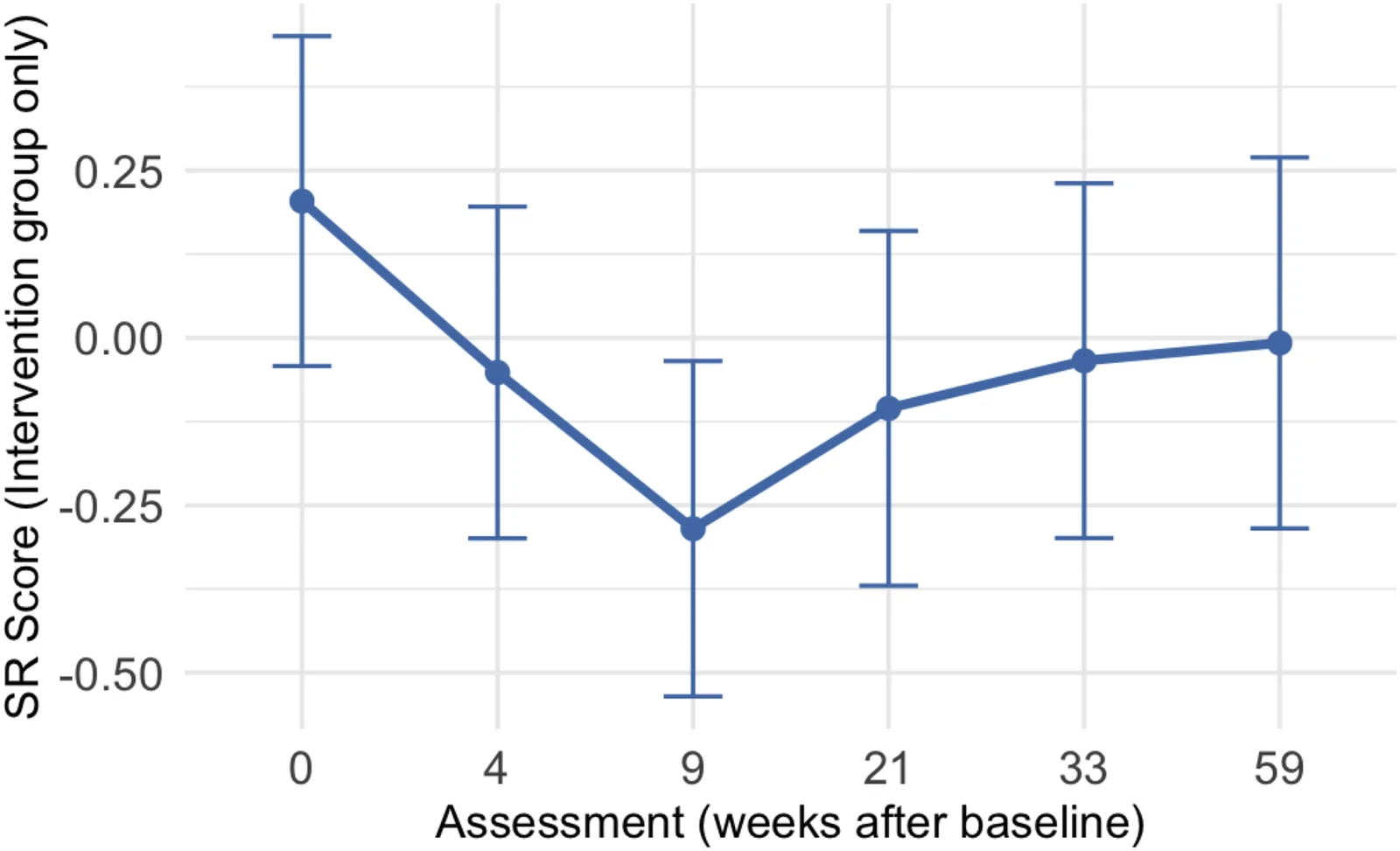
Development of SR scores in intervention group over assessments in the PP analysis. *Note.* Values represent model-based estimated marginal means (EMMs) from a LMM fitted to the intervention group only (PP analysis, *n* = 48). Error bars indicate 95% confidence intervals. The corresponding plot for the ITT analysis is presented in SM 7.

#### 3.2.3 H13–14: intervention effect on state self-compassion and state self-care

LMM analyses in the PP sample revealed a beneficial effect on state self-compassion for both the weekly and daily intervention exercises (H13; *b* = 0.59–0.69, 95% CI [0.35–0.97], both *p* < .001). State self-care also improved significantly following the weekly intervention (*b* = 0.81, 95% CI [0.40, 1.22], *p* < .001), whereas no statistically significant change was observed for the daily interventions (*p* = .240; see SM 8 for details). ITT analyses were not conducted for H13 and H14 because these hypotheses examined within-person changes associated with completed intervention exercises and therefore required corresponding pre- and post-exercise assessments.

#### 3.2.4 H15: dose-response analyses

The main dose-response analyses using robust linear regression provided limited evidence for associations between intervention dose and post-intervention outcomes. A significant positive association was observed only for the self-care subscale positive experience (H15; β = 0.30, *p* = .006). For all other outcomes, no significant linear dose–response relationships were found (all *p*s ≥ .131). Exploratory log-transformed models showed significant relationships for the self-care subscale positive experience (*p* = .043), and for the self-care subscale pacing (*p* = .044). Quadratic and threshold models did not yield stable results, see SM 9 for details.

#### 3.2.5 Exploratory moderation and dropout analyses

Exploratory moderation analyses revealed no evidence that baseline or post-intervention characteristics moderated changes from baseline to post-assessment (all *p*s ≥ .074). Specifically, optimism, positive appraisal style, internal locus of control, attitudes towards online interventions, program feedback, satisfaction with intervention, technical difficulties, age, and sex were not associated with differential changes in outcomes over time (details in SM 11).

As indicated above (see Section 3.1. Sample characteristics), completers and dropouts did not differ regarding baseline characteristics (i.e., attitudes towards online interventions (APOI), age or gender; all *p*s ≥ .202); but dropout differed significantly between groups, with higher dropout in IG compared to CG, *χ*^2^(1) = 7.57, *p* = .006.

#### 3.2.6 Sensitivity analyses

Sensitivity analyses – restricted to participants completing at least 14 intervention sessions as preregistered – yielded results consistent with the per-protocol analyses, with no meaningful changes in effect direction or significance (details in SM 10).

#### 3.2.7 Feedback on the intervention and assessment of potential harms

Satisfaction ratings among post-intervention assessment completers were high across all program dimensions (structure of content, content, handling of program and graphical representation). Detailed information on satisfaction is provided in SM 6.

Based on the INEP-ON, most participants reported positive effects, only few participants (15%) attributed any negative effect to the intervention, and these were mainly mild (see SM 13).

## 4 Discussion

### 4.1 Summary and interpretation of main findings

This RCT evaluated the effectiveness of *resiLIR HCP*, a self-guided online intervention targeting self-care and self-compassion, in promoting resilience among HCPs. Overall, the findings of the PP analysis indicate statistically significant group × time intervention effects for resilience and several mental health–related outcomes at post-intervention, whereas evidence for intervention effects was substantially weaker in the ITT analyses.

The intervention led to statistically significant improvements in the primary outcome, resilience, measured as a reduction in the stressor reactivity (SR) score at post-intervention in the PP analysis, indicating benefits among engaged participants with sufficient engagement. This effect was attenuated at follow-up and was not observed in ITT analyses. Thus, evidence for an intervention effect on the primary outcome was present in the PP but not in the ITT analysis.

In line with the conceptualization of the SR score as a dynamic indicator of individual stressor reactivity (Kalisch et al., 2021), within-group analyses in the PP sample revealed that reductions in SR score emerged already mid-intervention (t1) and were maintained until the 3-months follow-up (t3). Reductions at the 6-months (t4) and 12-months (t5) follow-up were no longer statistically significant. In the ITT analysis, none of the changes from baseline reached statistical significance. The PP findings therefore suggest short- to mid-term changes in stressor reactivity which gradually diminish over follow-ups (see Fig. 4) instead of transient fluctuations. Such patterns of attenuation over time are consistent with prior meta-analyses of resilience interventions (e.g., Schäfer et al., 2024b; Vanhove et al., 2016; Von Boros et al., 2026) and highlight the challenge of sustaining intervention effects, particularly in relatively healthy populations where ceiling effects may limit observable gains.

Resilience was also assessed using the BRS, which showed no statistically significant effect after adjustment for multiple testing. Differences between BRS and SR score reflect differences in their conceptualization of resilience (i.e., stressor reactivity vs. self-reported capacity of “bouncing back from adversity”) and their sensitivity to change, with the SR score capturing dynamic changes more adequately, whereas the BRS may be less sensitive to short-term effects, possibly because no specific reference period is provided. Notably, exploratory within-group analyses in the PP sample revealed significant and stable increases in BRS scores across follow-ups (see SM 12). However, these within-group changes should not be interpreted as evidence of an intervention effect due to the absence of a meaningful control group at follow-up assessments. Importantly, this is one of the first intervention studies so far using the SR score as outcome (e.g., Petri-Romão et al., 2025), following methodological recommendations (Chmitorz et al., 2018a; Schäfer et al., 2026) and thus making an important contribution to the operationalization and assessment of resilience beyond proxies such as self-perceived resilience. Differing patterns observed for the SR score and mental health symptoms further underline the importance of accounting for stressor exposure when evaluating intervention effects. Approaches relying solely on mental health symptom measures may overlook such differential patterns (Chmitorz et al., 2018a; Kalisch et al., 2020).

Significant intervention effects were also observed for self-care, self-compassion, perceived self-efficacy in dealing with stress, psychological distress, and depressive and anxiety symptoms in the PP analyses. These effects were strongest at post-intervention and partially sustained at follow-up. No significant intervention effects were found for work-related outcomes, i.e., subscales of burnout and work–life balance. In the ITT analyses, only psychological distress and the pacing subscale of self-care showed statistically significant effects, each limited to the post-intervention assessment. However, neither remained statistically significant after adjusting for multiple testing.

The contrast between PP and ITT findings may suggest the importance of intervention engagement for achieving beneficial outcomes; however, this pattern does not establish a causal effect of adherence. Also, the dose–response associations provided no conclusive evidence of a consistent relationship between intervention dose and outcomes. Associations were statistically significant only for the positive experience subscale of self-care in the linear robust analysis, and, additionally, for the pacing subscale of self-care in the exploratory logarithmic analysis, while finding no significant dose–response associations for resilience, self-compassion, and burnout. Therefore, the stronger effects observed in the PP analyses may also be attributed to other factors than intervention exposure. Participants who adhere to the intervention may differ from those who do not, for example with respect to motivation or available resources. However, beneficial effects were observed among participants who met the (predefined) adherence criterion, while the weaker ITT findings suggest that these effects did not generalize to the full randomized intervention group.

Interestingly, in the PP analyses, self-compassion continued to increase between post-intervention and follow-up. Similar evidence comes from a meta-analysis of mindfulness-based interventions, in which effects on self-compassion tended to be larger at follow-up than at post-intervention assessment, suggesting that changes in self-compassion may continue to unfold over time (Wasson et al., 2020). This notion is in line with conceptualizations of self-compassion as a trainable skill that may need some time to develop through ongoing practice (Neff and Germer, 2013). Consistent with this view, the inspection of within-group trajectories in the PP sample across all assessments (t0–t5) indicated that self-compassion increased steadily until the first follow-up, where it reached a plateau, whereas mindfulness exhibited a delayed increase that became apparent only at follow-up (see SM 12). Beyond these trait-like outcomes, pre-post assessments during the training exercises indicated immediate increases in state self-compassion for both weekly and daily interventions, and in state self-care after the weekly interventions. This pattern suggests that even brief intervention components may produce meaningful state-level changes. Such increases in self-compassion are relevant, as repeated state changes may represent a potential pathway through which longer-term improvements emerge (e.g., Mey et al., 2023). For state self-care, the weekly training interventions may have promoted momentary perceived self-care by allowing reflection and planning, whereas brief daily interventions may have been less conducive to immediate evaluative shifts, as they can highlight limited opportunities for self-care on a given day rather than foster positive reappraisal. Little is currently known about the underlying change processes and mechanisms, emphasizing the potential of future studies using ecological momentary assessment to capture these dynamics in daily life (e.g., Mey et al., 2023; Schäfer et al., 2026).

Regarding work-related outcomes, i.e., work-life balance as well as burnout with its subscales emotional exhaustion, depersonalization and personal accomplishment, no significant intervention effects were observed. This aligns with prior research indicating that changes in occupational outcomes are more likely driven by structural changes at the workplace than interventions targeting only the individual level. Psychological distress and occupational outcomes among HCPs arise from reciprocal interactions between individuals and multiple environmental levels (Almeida-Meza et al., 2025). Burnout is strongly shaped by organizational and structural factors such as workload and working conditions (Robertson et al., 2016), which may constrain the impact of interventions solely focusing on the individual. Accordingly, organizational approaches – such as improving working conditions (e.g., flexible scheduling) – appear particularly promising in alleviating HCPs' burden and improving work-life balance (Aust et al., 2024), and have been shown to be more effective than individual-level strategies in reducing burnout (West et al., 2016). Extending this evidence, a recent review found that multi-level interventions combining individual and organizational strategies provide the most robust evidence for burnout reduction (Bagasi et al., 2025). Consequently, mental health promotion for HCPs should combine individual, (digital) resilience interventions with strategies addressing organizational factors.

### 4.2 Engagement, adherence and user satisfaction

Meaningful improvements in the present study were primarily observed in the PP analyses, whereas evidence for intervention effects was limited in the ITT analyses. Overall adherence was limited (only 43.2% meeting the predefined adherence criterion). Low adherence is a well-documented challenge in self-guided digital mental health interventions (Eysenbach, 2005), with similar patterns observed in other studies (Forbes et al., 2023; Hersch et al., 2016). Comparisons across studies remain difficult due to heterogeneous definitions of adherence (Valentine et al., 2025).

Real-world data suggest even lower engagement with freely available mental health apps, with median 15- and 30-day retention rates below 4% (Baumel et al., 2019). Among HCPs, lack of time and unwillingness to engage with work-related interventions during leisure time can be barriers, whereas adaptive “just-in-time” notifications (e.g., prompts triggered by arriving at work) and human support may facilitate engagement (Zhang et al., 2025).

In the present study, allowing participants to select the timing of reminders represented an initial step towards personalization; however, some participants expressed a desire for greater flexibility. In addition, the mode of delivery may have hindered engagement. The intervention was deliberately designed as a web-based program accessed via an internet browser, assuming that downloading an app might represent a barrier. However, these design choices may also have constituted a hindrance for others, as users are increasingly accustomed to mobile apps. Moreover, parts of the intervention were optimized for larger screens which may have reduced usability on smartphones. Future iterations may benefit from a mobile-first design approach. Finally, engagement may also have been affected by procedural requirements: for data protection, participants were required to authenticate twice on the platform.

User satisfaction among participants was high across content, structure, and design aspects, suggesting that the intervention was highly acceptable and perceived as relevant. However, this feedback should be interpreted with caution due to potential selection bias, as participants who provided feedback were also those who showed higher engagement with the intervention (see SM 6). Importantly, prior evidence suggests that user experience ratings are not significantly correlated with actual app usage (Kaveladze et al., 2022), underscoring the need for qualitative research examining the mechanisms of engagement in digital interventions.

Overall, while self-guided digital interventions offer flexibility, scalability, and low-threshold access, limited adherence remains a challenge. Guided, blended, or organizationally embedded intervention formats may therefore offer advantages, particularly for burdened populations like HCPs, by providing additional structure, human support, and opportunities to engage during working hours (Zhang et al., 2025). Sustainable improvements – particularly in work-related outcomes – likely require integration into broader, multi-level prevention strategies also addressing structural demands (e.g., Aust et al., 2024; West et al., 2016).

### 4.3 Strengths and limitations

Several strengths of this study should be acknowledged. First, resilience as primary outcome was operationalized using the SR score (Kalisch et al., 2020), a conceptually grounded and methodologically robust approach accounting for individual differences in stressor exposure. In addition, the assessment of multiple mental health, resilience-related, and work-related outcomes provides a comprehensive picture of intervention effects. Finally, multiple follow-up assessments over 12 months allow for insights into post-intervention outcome trajectories.

However, several limitations should also be considered. Overall, adherence to the self-guided intervention was limited, and intervention effects differed substantially between the PP and ITT analyses. The stronger effects observed in the PP analyses may partly reflect differential attrition, missing data, or potential differences between adherent and non-adherent participants with respect to non-assessed variables (e.g., motivation, personal resources). Therefore, the stronger effects observed in the PP analyses cannot be interpreted as robust evidence that greater intervention exposure causes greater improvements. PP analyses relied on a reduced and more imbalanced sample than anticipated (CG: *n* = 84; IG: *n* = 45), potentially reducing statistical power for detecting small effects, which are common for digital resilience interventions (Schäfer et al., 2024b). Consequently, after adjustment for multiple testing, effects on depressive and anxiety symptoms in the PP analyses were no longer significant. Depressive and anxiety symptoms were analyzed both as components of the SR score and as separate outcomes. While this may appear redundant, it allows comparability with other intervention studies and the possibility to include our findings in future meta-analyses, given that the SR score is not yet widely used and intervention effects are typically evaluated using post-intervention mental health outcomes (e.g., Kunzler et al., 2020a, Kunzler et al., 2020b; Liu et al., 2020; Schäfer et al., 2026). Moreover, recent research has raised concerns regarding the measurement invariance and comparability of the PHQ-9 across different populations (Hlynsson et al., 2025). As PHQ-9 scores from sister trial samples (students and the general population) contributed to the normative reference used to calculate SR scores, we conducted a sensitivity analysis in which the SR score was calculated using the HCP sample only. This analysis yielded comparable effect sizes and directions of effects (see SM 14). Moreover, all outcomes were based on self-report measures. While most instruments were well-established and validated, state measures of self-care and self-compassion were assessed using non-validated single-item measures, warranting cautious interpretation. Furthermore, due to the nature of the study, participants could not be blinded to group allocation, and the use of a waitlist control condition does not control for expectancy effects. Thus, positive expectancies may provide an alternative explanation for some of the observed effects. Future studies could address this limitation by using active control groups.

Although the intervention was built on evidence-based resilience factors and informally reviewed by associated HCPs, the target population was not systematically involved in the development and usability testing of the intervention. Future studies should adopt a more systematic and iterative co-design approach, involving HCPs early and repeatedly to evaluate and refine both intervention content and usability prior to implementation (Roennfeldt et al., 2026; Schäfer et al., 2026). Finally, the study relied on a self-selected sample, limiting representativeness and generalizability.

## 5 Conclusion

In this RCT, PP analyses indicated that *resiLIR HCP*, a self-guided, web-based resilience intervention for healthcare professionals focusing on self-care and self-compassion, showed significant between-group improvements in resilience (SR score) and beneficial intervention effects on self-care, self-compassion, and overall psychological distress. Effects on depressive and anxiety symptoms were less robust in the PP analyses and not statistically significant after adjustment for multiple testing. However, these findings were not supported by the ITT analyses. No statistically significant intervention effect was found for the primary resilience outcome, and none of the secondary outcome effects remained statistically significant in the ITT analyses after adjustment for multiple testing. Limited adherence and the absence of effects on work-related outcomes highlight challenges in sustaining engagement with the intervention and achieving impact on occupational outcomes through self-guided digital interventions alone. Future research should focus on enhancing engagement and integrating digital resilience interventions into organizational prevention strategies to alleviate challenges associated with the healthcare workforce crisis.

## Informed consent

All participants gave their written consent in accordance with the Declaration of Helsinki. Privacy rights of human subjects have been observed.

## Ethical approval

The study was approved by the Ethics Committee of the State Medical Association of Rhineland-Palatinate, Germany (no. 2022-16888).

## Declaration of Generative AI and AI-assisted technologies in the writing process

During the preparation of this work the authors used ChatGPT to refine language in the manuscript and for adaptation and troubleshooting of R code. After using this tool, the authors reviewed and edited the content as needed and take full responsibility for the content of the published article.

## Funding

This work was supported by the European Funds for Regional Development (REACT-EU) with the Ministry of Science and Health of Rhineland-Palatinate (grant number 84009588) and the European Union within the Horizon Europe Program: XR2ESILIENCE (grant 101137237).

## Data and code availability statement

The preregistration and R code used for the analyses are available on Open Science Framework: https://osf.io/vp4ms. Individual participant data are not publicly available but may be obtained in anonymized form from the corresponding author upon reasonable request.

## Declaration of competing interest

The authors declare that they have no known competing financial interests or personal relationships that could have appeared to influence the work reported in this paper.
