# Supplementary material for: resiLIR HCP: A self-guided digital resilience intervention for healthcare professionals focusing on self-care and self-compassion – A randomized controlled trial
Source: Internet Interv. 2026 Oct 6;46:101010. doi: 10.1016/j.invent.2026.101010 (PMC13670936; doi:10.1016/j.invent.2026.101010)
Supplement: Supplementary file 1 [file mmc1.pdf]

Supplementary Material for Manuscript

resiLIR HCP: A self-guided digital resilience intervention for healthcare professionals focusing on self-care and self-compassion – A randomized controlled trial

Authors: Lisa von Boros, Sarah K. Schäfer, Tabea Werner, Carolina Pank, Friederike Köhler, Federico Zappalà, Benedikt E. Wirth, Tobias Zimmermann, Michael Moos, Lars Gottfriedsen, Alexander Scherrer, Isabella Helmreich, Klaus Lieb, Michèle Wessa, and Oliver Tüscher

Corresponding author: Prof. Dr. Sarah K. Schäfer; e-mail: sarah.schaefer@lir-mainz.de

Table of content

1. Time plan of assessments 1

2. Deviations from preregistration 2

3. Detailed description of measures 4

4. Baseline characteristics for PP sample 7

5. Descriptive statistics of PP sample for IG and CG at t0, t2 and t3 8

6. Analysis of usage, satisfaction and adherence 9

7. ITT plots for H1-12 10

8. Details of results for H13 and H14 12

9. Analyses for H15 (dose-response analyses) 13

Main analysis: Linear dose–response models using robust regression 13

Sensitivity analysis: Linear dose–response models using ordinary least squares (OLS) 13

Exploratory analysis: Logarithmic dose-response models using robust regression 14

10. Sensitivity analyses for H1-12 and H15 15

Per protocol analyses with participants completing minimum 14 interventions 15

11. Moderator and dropout analyses 17

12. Exploratory within-group analyses for self-compassion, mindfulness and resilience (BRS) 18

13. Assessment of potential negative effects of the intervention (INEP-ON) 21

14. Sensitivity analysis of H1 using an SR Score based on an HCP-only normative regression 26

15. References in Supplementary Material 27

1. Time plan of assessments

Table SM 1. **Instruments collected over study time in assessments via SosciSurvey**

**Questionnaire**	**t0**	**t1**	**t2**	**t3**	**t4**	**t5**	**IG only**
Weeks after baseline	*0*	*4*	*9*	*21*	*33*	*59*	
Brief Resilience Scale (BRS; Kunzler et al., 2018)	X	X	X	X	X	X	
General Health Questionnaire–12 (GHQ-12; Schrnitz et al., 1999)	X	X	X	X	X	X	
Self-Compassion Scale (SCS; Hupfeld & Ruffieux, 2011)	X	X	X	X	X	X	
Hamburg Self-Care Survey (HSF; Harfst et al., 2009)	X	X	X	X	X	X	
Mindful Awareness and Attention Scale – Short Form (MAAS; Höfling et al., 2011)	X	X	X	X	X	X	
Perceived Stress Scale 2&2 (PSS 2&2; Schäfer et al., 2023)	X	X	X	X	X	X	
Generalized Anxiety Disorder Scale (GAD-7; Spitzer et al., 2006)	X	X	X	X	X	X	
Patient Health Questionnaire–9 (PHQ-9; Löwe et al., 2002)	X	X	X	X	X	X	
Trier Short Scale for the Assessment of Work–Life Balance (TKS-WLB; Syrek et al., 2011)	X	X	X	X	X	X	
Maslach Burnout Inventory (MBI; Büssing & Perrar, 1992)	X	X	X	X	X	X	
Mainz Inventory of Microstressors (MIMIS; Chmitorz et al., 2020)	X	X	X	X	X	X	
Optimism–Pessimism Scale (SOP-2; Kemper et al., 2014)	X		X	X	X	X	
Positive Appraisal Style Scale – Process (PASS-process; Petri-Romão et al., 2021)	X		X	X	X	X	
Internal–External Locus of Control Short Scale (IE-4; Kovaleva et al., 2014)	X		X	X	X	X	
General Self-Efficacy Short Scale (ASKU; Beierlein et al., 2014)	X		X	X	X	X	
Kessler Psychological Distress Scale (K-10; Kessler et al., 2003)	X		X	X	X	X	
Attitudes toward Psychological Online Interventions (APOI; Schröder et al., 2015)	X						
Inventory for the Balanced Assessment of Negative Effects of Online Interventions (INEP-ON; Ladwig et al., 2014)				X			X
Client Satisfaction Questionnaire – Intervention (CSQ-I; Boß et al., 2016)				X			X
Feedback items on intervention				X			X
Technical difficulties				X			X

Note. X indicates when the respective scale was assessed. IG only = only assessed in intervention group; not control group.

2. Deviations from preregistration

Table SM 2. **Deviations from preregistration.** Preregistration available here: https://clinicaltrials.gov/study/NCT05812716

	Preregistration	Final study / final manuscript
Hypotheses (H14)	Self-care interventions (weekly intervention and daily training intervention) in the practice modules: A) For the weekly interventions, the state self-compassion measure will be significantly higher after the interventions (comparison of $st_{1.1pre}$ vs. $St_{1.1post}$ and $st_{3.1pre}$ vs. $st_{3.1post}$). B) For the first session of the daily training interventions, the state self-compassion measure will be significantly higher after the interventions (comparison of $st_{1.2pre}$ vs. $St_{1.2post}$ and $st_{3.2pre}$ vs. $st_{3.2post}$).	There was a mistake in our preregistration, so that we mistakenly referred to state self-compassion as outcome for the exercises which focused on self-care. The correct formulation of H14 would be: Self-care interventions (weekly intervention and daily training intervention) in the practice modules: A) For the weekly interventions, the **state self-care measure** will be significantly higher after the interventions (comparison of $st_{1.1pre}$ vs. $St_{1.1post}$ and $st_{3.1pre}$ vs. $st_{3.1post}$). B) For the first session of the daily training interventions, the **state self-care measure** will be significantly higher after the interventions (comparison of $st_{1.2pre}$ vs. $St_{1.2post}$ and $st_{3.2pre}$ vs. $st_{3.2post}$).
Data analysis (H1 to H12)	The analysis plan for H1 to H11 is as follows:<br>Differences between study groups in primary and secondary outcomes before (t0) and after the intervention (t2) and at the time of the first follow-up (t3) will be examined **using mixed analyses of covariance (ANCOVA),** with group (intervention vs. waitlist-control) as a between-subjects factor, time (t0, t2, t3) as a within-subjects factor and pre-measurement (t0) included as a covariate to increase test power. Outcomes will be the primary outcome resilience (measured with SR Score) and the secondary outcomes resilience (measured with BRS), perceived stress, self-care, self-compassion, mindfulness, mental health, depression, anxiety, burnout, and work-life-balance, respectively.<br><br>The analysis plan for H12 is as follows: term effect over the follow-up measurements (t0 to t5) within the intervention group will be tested using a repeated measures ANOVA.<br>• If applicable, post-hoc t-tests and planned contrasts will be conducted.<br>• Effect sizes will be calculated.<br>• Eta2 will be calculated as measure for effect size for the ANOVA.	We conducted linear mixed models instead of mixed ANCOVAs as this approach imposes strict assumptions for repeated-measures data—such as complete data at all time points, sphericity, independence of residuals, and homogeneous regression slopes—which were not met in our dataset.<br><br>Instead, following Twisk's (2021) recommendation, we applied **linear mixed-effects models with random intercepts to account for within-person dependence and to allow unbiased baseline adjustment**. We estimated both an **overall intervention effect** across post-baseline assessments and **time-specific effects at week 9 and week 21** using dummy-coded follow-up indicators.
Data analysis	The analysis plan for H13 and H14 is as follows:	**We used linear mixed-effects models with random intercepts for participants**. This

(H13 to H14)	• In order to test whether the changes in the state measurements (pre- to post-intervention in state self-compassion and state self-care) are significant, we will conduct within-group t-tests. We will perform separate tests for state self-compassion and state self-care, each for (1) the weekly training interventions and (2) the first practice of daily interventions, respectively.	approach accounts for the nested data structure (repeated pre–post measurements within individuals), and handles unequal numbers of observations and missing data more appropriately. Therefore, mixed models were used instead of separate paired t-tests.
Power analysis	… the sample size design was calculated with a medium effect size, a significance level of 0.05 and a power of 0.90 using G*Power (Faul et al., 2007). This results in a required sample size of *n* = 74 per group (*N* = 146).	The preregistered **power analysis was based on the assumption that the data would be analyzed using ANCOVAs**. In the final analyses, linear mixed-effects models were used instead, as they better accounted for the structure of the data.<br><br>The preregistration reported *n* = 74 per group and *N* = 146, which are arithmetically inconsistent. The present manuscript was therefore corrected and now refers to the preregistered total sample-size target (*N* = 146) and respective groups (*n* = 73).

3. Detailed description of measures

Resilience approximated with the BRS

The 6-item Brief Resilience Scale (BRS; Chmitorz, Wenzel, et al., 2018) was used as a self-report measure to approximate resilient outcomes. Participants rated their agreement with statements on post-stressor recovery in challenging situations on a 5-point Likert scale ranging from 1 (strongly disagree) to 5 (strongly agree). After reverse-coding three items, higher total scores indicate greater resilience. Internal consistency in the present sample was good ($\alpha = 0.84$, $\omega = 0.88$).

Psychological distress

Mental health was operationalized as the absence of psychological distress and measured with the 12-item General Health Questionnaire (GHQ-12; Schrnitz et al., 1999). Participants indicated the extent to which they had experienced health-related difficulties in recent weeks. Items were rated on a 4-point Likert scale ranging from 1 (not at all / better than usual) to 4 (much more than usual / worse than usual), with six items being reverse-coded. Items were summed to yield a total score, with higher values indicating greater psychological distress. Internal consistency was good (α / $\omega = 0.87$).

Self-Compassion

Self-compassion was assessed with the 26-item Self-Compassion Scale (SCS; Hupfeld & Ruffieux, 2011). Participants rated how they typically respond to themselves in challenging situations on a 5-point Likert scale from 1 (almost never) to 5 (almost always). After reverse-coding negatively phrased items, a mean total score was calculated, with higher values indicating greater self-compassion. Internal consistency was excellent in the present sample ($\alpha = 0.94$, $\omega = 0.95$).

Self-Care

Self-care was measured with the Hamburg Self-Care Survey (HSF; Harfst et al., 2009). The questionnaire consists of two subscales, i.e., positive experience and pacing. Positive experience refers to the ability to accept and enjoy positive experiences and behaviors while pacing focuses on mindful self-management and awareness of one's own limits. The survey assesses the extent to which individuals engaged in self-care behaviors during the past four weeks (e.g., taking breaks). Items are rated on a 5-point Likert scale

ranging from 1 (not at all applicable) to 5 (very applicable). For each subscale, a mean score was calculated, with higher values indicating greater self-care. Internal consistency was excellent in the present sample for both subscales (α = 0.90–0.93, ω = 0.93–0.95).

Mindfulness

Mindfulness was assessed using the 10-item Mindful Awareness and Attention Scale-Short Form (MAAS; Höfling et al., 2011). The scale measures the frequency of mindful versus mindless states in everyday life, with statements being rated on a 6-point Likert scale ranging from 1 (almost always) to 6 (almost never). After reverse-coding negatively phrased items, a mean score was calculated. Higher values indicate greater mindfulness. Internal consistency in the present sample was excellent (α = 0.92, ω = 0.94).

Perceived Stress

Perceived stress was assessed using the Perceived Stress Scale 2&2 (PSS 2&2; Schäfer et al., 2023), which comprises two 2-item subscales measuring helplessness and self-efficacy. Participants rated how often they had experienced stress-related thoughts and feelings during the past month on a 5-point Likert scale from 1 (never) to 5 (very often). Mean scores were computed for each subscale; higher helplessness scores indicate greater perceived stress, whereas higher self-efficacy scores indicate lower perceived stress. Spearman–Brown (SB) coefficients (recommended as the most appropriate measure for internal consistency for two-item scales; Eisinga et al., 2013) were acceptable for helplessness (SB = 0.67) and good for self-efficacy (SB = 0.75).

Anxiety symptoms

Anxiety symptoms were measured using the 7-item Measurement for Generalized Anxiety Disorder (GAD-7; Spitzer et al., 2006). Participants rated the extent to which they had been affected by various anxiety-related symptoms over the past two weeks (e.g., restlessness) on a 4-point Likert scale ranging from 1 (not at all) to 4 (nearly every day). A sum score was calculated, with higher values indicating greater anxiety. Internal consistency in the present sample was good (α = 0.83, ω = 0.88). Total scores range from 0 to 21, with established cut-offs of 5, 10, and 15 indicating mild, moderate, and severe anxiety, respectively (Spitzer et al., 2006).

Depressive symptoms

Depressive symptoms were measured with the 9-item Patient Health Questionnaire (PHQ-9; Löwe et al., 2002). Participants rated the extent to which they had been affected by depressive symptoms (e.g., hopelessness) over the past two weeks on a 4-point Likert scale ranging from 1 (not at all) to 4 (nearly every day). A sum score was calculated, with higher values indicating more severe depressive symptoms. Internal consistency in the present sample was good (α = 0.83, ω = 0.86). Established cut-offs of 5, 10, 15, and 20 indicate mild, moderate, moderately severe, and severe depressive symptoms, respectively (Kroenke et al., 2001).

Work-life balance

Work-life balance was assessed with the 5-item Trier Short Scale for the Assessment of Work-Life Balance (TKS-WLB; Syrek et al., 2011). Participants rated the extent to which statements applied to them on a 5-point Likert scale ranging from 1 (does not apply at all) to 5 (fully applies). A mean score was calculated, with higher values indicating a better work-life balance. Internal consistency in the present sample was good to excellent (α = 0.86, ω = 0.90).

Burnout

Burnout was assessed using the 25-item Maslach Burnout Inventory (MBI; Büssing & Perrar, 1992), which comprises three subscales: emotional exhaustion, depersonalization, and personal accomplishment. Participants rate statements about their feelings and experiences at work on a 7-point Likert scale from 1 (never) to 7 (every day). For emotional exhaustion and depersonalization higher scores indicate more burnout; whereas for personal accomplishment lower scores indicate more severe burnout. Items were aggregated separately for each subscale. Internal consistencies for all subscales were good to excellent (α = 0.82–0.91, ω = 0.85–0.93).

Stressor exposure

We assessed exposure to micro stressors using the Mainz Inventory of Micro Stressors (MIMIS; Chmitorz et al., 2020). Participants were presented with a list of 58 potentially stressful everyday situations and indicated on how many days (1 to 7 or "did not occur") the respective situation had occurred during the past 7 days. As the instrument represents a checklist of unrelated stressor events rather than a unidimensional latent construct, internal consistency indices (α / ω) were not calculated.

State self-compassion and state self-care

Momentary self-compassion and self-care were assessed using two single-item visual analogue scales administered immediately before and after the weekly and daily training interventions. Participants rated how self-compassionate and self-caring they felt in the moment – that is, the extent to which they perceived themselves as treating themselves in a supportive and caring manner – on a 10-point scale ranging from 1 (not at all) to 10 (very much). Higher values indicate greater state self-compassion and state self-care.

4. Baseline characteristics for PP sample

Table SM 4. Sample characteristics for PP sample at baseline.

	**All (*n* = 129)**	**Control (*n* = 84)**	**Intervention (*n* = 45)**
**Age: *M* (*SD*)**	42.09 (12.33)	41.05 (11.66)	44.04 (13.42)
**Sex: # (%)**			
Women	105 (81.4%)	68 (81.0%)	37 (82.2%)
Men	24 (18.6%)	16 (19.0%)	8 (17.8%)
**Education (attended years of school): # (%)**			
Up to 9 years	3 (2.3%)	2 (2.4%)	1 (2.2%)
10 – 11 years	31 (24.0%)	21 (25.0%)	10 (22.2%)
12+ years	95 (73.6%)	61 (72.6%)	34 (75.6%)
**Profession: # (%)**			
Physician	12 (9.3%)	8 (9.5%)	4 (8.9%)
Nurse	23 (17.8%)	17 (20.2%)	6 (13.3%)
Physical therapist	23 (17.8%)	14 (16.7%)	9 (20.0%)
Speech therapist	30 (23.3%)	17 (20.2%)	13 (28.9%)
Psychotherapist	10 (7.8%)	7 (8.3%)	3 (6.7%)
Dentist	3 (2.3%)	1 (1.2%)	2 (4.4%)
Medical technical or laboratory assistant	1 (0.8%)	1 (1.2%)	–
Geriatric nurse	2 (1.6%)	2 (2.4%)	–
Other	25 (19.4%)	17 (20.2%)	8 (17.8%)

Note. M = mean, SD = standard deviation, # = number, PP = per protocol. For the PP analysis, participants in the intervention group were required to have completed at least 10 intervention sessions to ensure sufficient intervention exposure. Additionally, participants in both groups were required to have provided at least one post-baseline assessment of the primary outcome (SR Score), either at post-intervention or at the 3-month follow-up. Here, PP criteria were applied at the analysis stage (e.g., also excluding participants' data when it failed the quality check, i.e., extremely fast responding indicated by TIME_RSI > 2).

5. Descriptive statistics of PP sample for IG and CG at t0, t2 and t3

Table SM 5. Descriptive statistics (means and standard deviations) of PP sample for IG and CG at baseline (t0), post-intervention (t2), and 3-month follow-up (t3).

	**Baseline (t0)**			**Post-intervention (t2)**			**3-month follow-up (t3)**		
**Group**<br>**Outcome**	**Total (n=129)**<br>***M* (*SD*)**	**CG (n=84)**<br>***M* (*SD*)**	**IG (n=45)**<br>***M* (*SD*)**	**Total (n=125)**<br>***M* (*SD*)**	**CG (n=80)**<br>***M* (*SD*)**	**IG (n=45)**<br>***M* (*SD*)**	**Total (n=110)**<br>***M* (*SD*)**	**CG (n=74)**<br>***M* (*SD*)**	**IG (n=36)**<br>***M* (*SD*)**
Resilience (SR Score)	0.07 (0.83)	0.00 (0.78)	0.20 (0.89)	−0.12 (0.81)	−0.03 (0.83)	−0.28 (0.76)	−0.06 (0.79)	−0.04 (0.79)	−0.11 (0.79)
Resilience (BRS)	3.36 (0.77)	3.39 (0.77)	3.31 (0.79)	3.60 (0.69)	3.56 (0.67)	3.66 (0.72)	3.57 (0.75)	3.52 (0.76)	3.68 (0.71)
Self-care – Positive experience	4.20 (0.77)	4.19 (0.77)	4.21 (0.78)	4.29 (0.76)	4.20 (0.83)	4.46 (0.59)	4.30 (0.74)	4.24 (0.75)	4.44 (0.69)
Self-care - Pacing	3.48 (0.83)	3.50 (0.86)	3.43 (0.79)	3.57 (0.87)	3.36 (0.90)	3.96 (0.66)	3.71 (0.81)	3.60 (0.85)	3.94 (0.69)
Self-compassion	3.25 (0.62)	3.23 (0.62)	3.27 (0.64)	3.42 (0.66)	3.36 (0.69)	3.54 (0.57)	3.45 (0.64)	3.34 (0.65)	3.69 (0.56)
Mindfulness	4.09 (0.78)	4.11 (0.76)	4.06 (0.83)	4.06 (0.84)	4.06 (0.88)	4.06 (0.75)	4.25 (0.82)	4.19 (0.83)	4.39 (0.80)
Perceived stress –Helplessness	3.09 (0.78)	3.07 (0.79)	3.12 (0.78)	2.86 (0.81)	2.93 (0.84)	2.73 (0.74)	2.81 (0.72)	2.79 (0.73)	2.86 (0.69)
Perceived stress –Self-efficacy	3.84 (0.68)	3.89 (0.66)	3.74 (0.71)	3.96 (0.63)	3.84 (0.65)	4.16 (0.54)	3.90 (0.66)	3.86 (0.69)	3.97 (0.60)
Psychological distress	12.08 (5.03)	11.77 (5.18)	12.64 (4.74)	10.40 (5.25)	11.65 (5.37)	8.18 (4.23)	10.55 (5.45)	11.41 (5.78)	8.78 (4.26)
Depressive symptoms	15.00 (4.11)	14.87 (4.16)	15.24 (4.06)	14.28 (4.14)	14.71 (4.52)	13.51 (3.28)	14.08 (3.73)	14.26 (3.83)	13.72 (3.54)
Anxiety symptoms	12.68 (3.66)	12.23 (3.43)	13.53 (3.96)	11.81 (3.34)	12.09 (3.68)	11.31 (2.60)	11.72 (3.36)	11.84 (3.47)	11.47 (3.15)
Burnout –Emotional exhaustion	3.06 (1.17)	2.95 (1.12)	3.26 (1.25)	3.11 (1.29)	3.05 (1.21)	3.22 (1.43)	2.98 (1.28)	3.00 (1.28)	2.94 (1.28)
Burnout –Depersonalisation	1.97 (1.17)	1.86 (1.05)	2.19 (1.35)	2.22 (1.33)	2.17 (1.21)	2.30 (1.53)	2.08 (1.20)	2.12 (1.22)	1.99 (1.16)
Burnout –Personal accomplishment	5.93 (0.77)	5.93 (0.74)	5.92 (0.82)	5.91 (0.81)	5.87 (0.85)	5.98 (0.73)	5.89 (0.82)	5.84 (0.86)	6.00 (0.75)
Work-life balance	3.39 (0.94)	3.45 (0.89)	3.27 (1.02)	3.45 (0.96)	3.51 (0.95)	3.33 (0.98)	3.48 (0.93)	3.49 (0.93)	3.47 (0.95)

Note. Descriptive statistics are based on available per-protocol data at each assessment point; sample sizes therefore vary across time points due to missing data. CG = control group; IG = intervention group; PP = per-protocol.

6. Analysis of usage, satisfaction and adherence

Table SM 6a. Analysis of usage of *resiLIR HCP* x completion of post-intervention assessment

Outcome	All Intervention Users who logged into the platform at least once (*n* = 101)	Intervention Users Completing the Post-Intervention Assessment (*n* = 63)	Intervention Users Not Completing the Post-Intervention Assessment (*n* = 38)
Total time (in minutes) spent with intervention: *M (SD)*	178 (169)	242 (173)	71 (93)
Number of completed training interventions: *M (SD)*	10.01 (9.53)	14.59 (8.62)	2.42 (5.11)
Satisfaction with structure of content: *M (SD)*	n/a	4.19 (0.78)	n/a
Satisfaction with content itself: *M (SD)*	n/a	4.24 (0.84)	n/a
Satisfaction with handling of program: *M (SD)*	n/a	3.90 (1.11)	n/a
Satisfaction with graphical representation: *M (SD)*	n/a	4.38 (0.79)	n/a

Note. Intervention users here are defined as participants who logged at least once into the platform, irrespective of the number of modules and training interventions completed. *n* = 17 users (of the *n* = 118 users randomized into the intervention group) never logged into the platform and are therefore not included in this analysis. *M* = mean*, SD* = standard deviation.

Table SM 6b. Adherence to the intervention

Adherence group	Definition	*n* (of total *n* = 118 in intervention group)	% of intervention group
**No engagement in intervention**	No login to platform and/or no intervention content accessed	36	30.5
**Theory-only users**	Started and/or completed theoretical modules only, without engaging in any practical training modules	15	12.7
**Minimal or low adherence**	Completed 1–9 daily/weekly training interventions	16	13.6
**Sufficient or high adherence**	Completed ≥10 daily/weekly training interventions	51	43.2

Note. Adherence was defined based on intervention usage and was independent of assessment completion; thus, per-protocol analyses included fewer participants than indicated by adherence categories.

7. ITT plots for H1-12

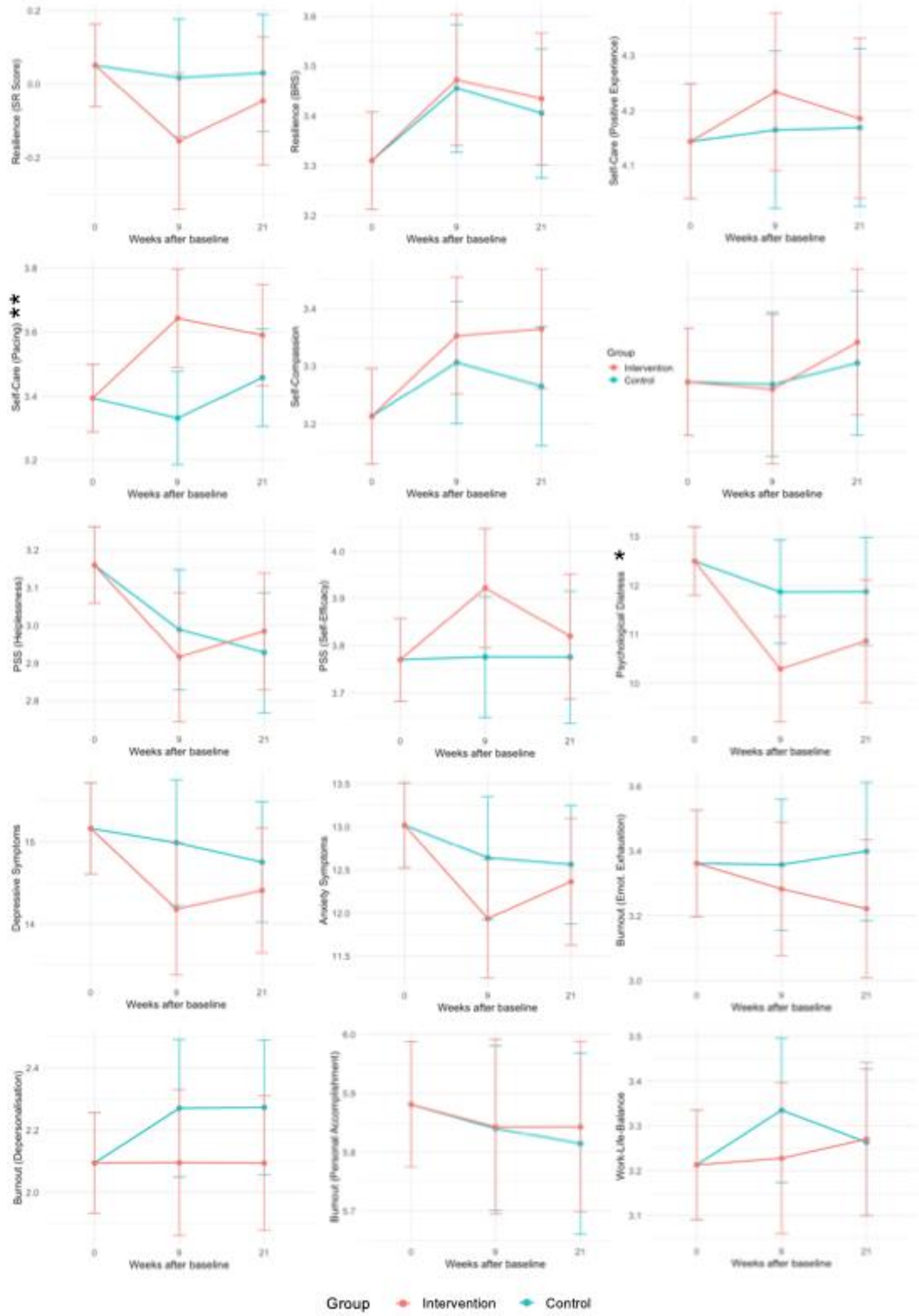

Figure SM 7a. Plots of H1 to H11 (intention-to-treat analyses)
Note. Values represent model-based estimated marginal means (EMMs) from a LMM adjusted for baseline. Error bars show 95% confidence intervals. Significant overall group x time interactions (according to raw *p*-values, not Hommel-adjusted *p*-values) are indicated by asterisks in the panels (*: $p < .05$, **: $p < .01$)

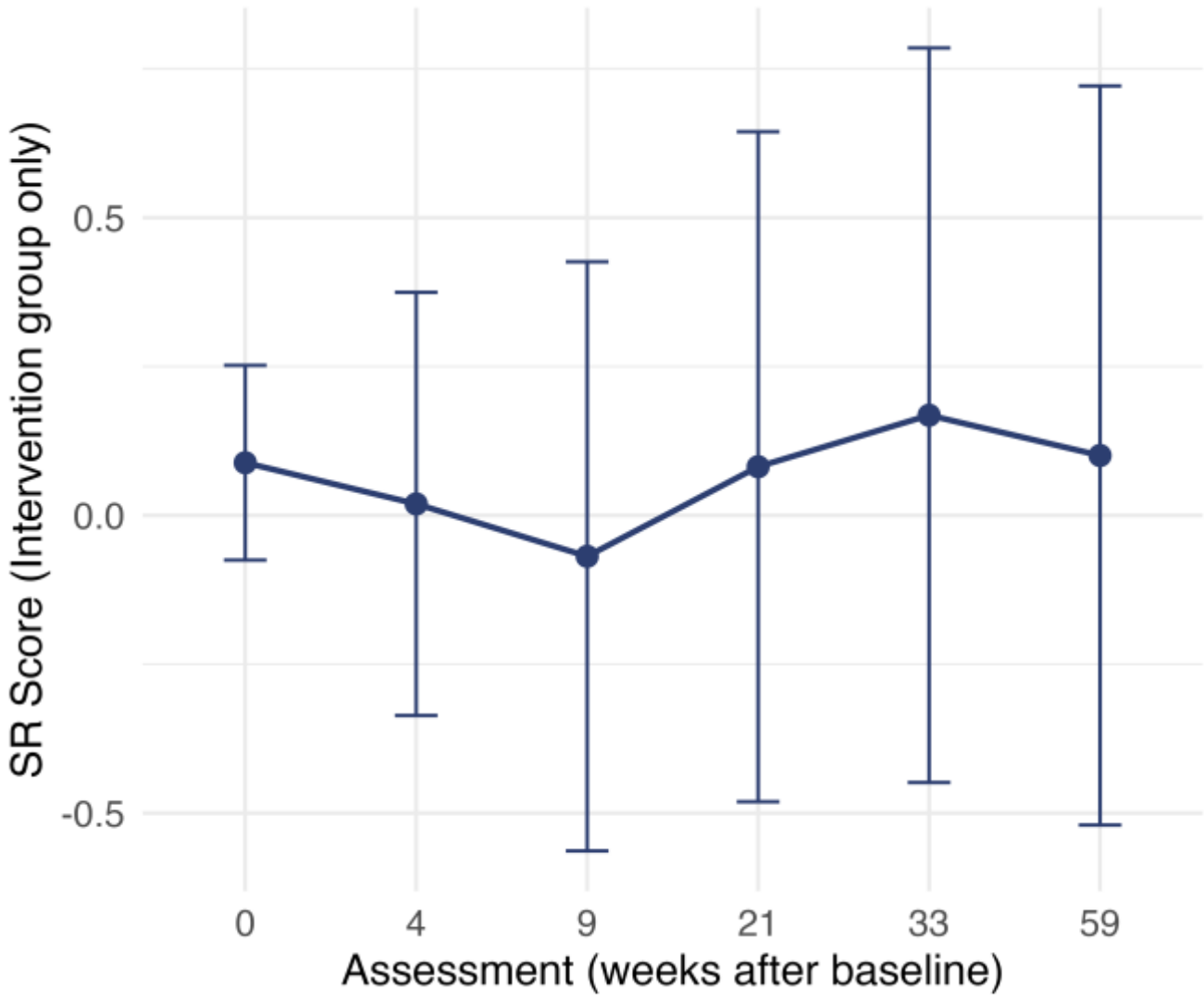

Figure SM 7b. Development of SR scores over assessments in the intervention group (intention-to-treat analysis).

Note. Values represent model-based estimated marginal means (EMMs) from linear mixed models fitted to the intervention group only and pooled across multiply imputed datasets. Error bars indicate 95% confidence intervals.

8. Details of results for H13 and H14

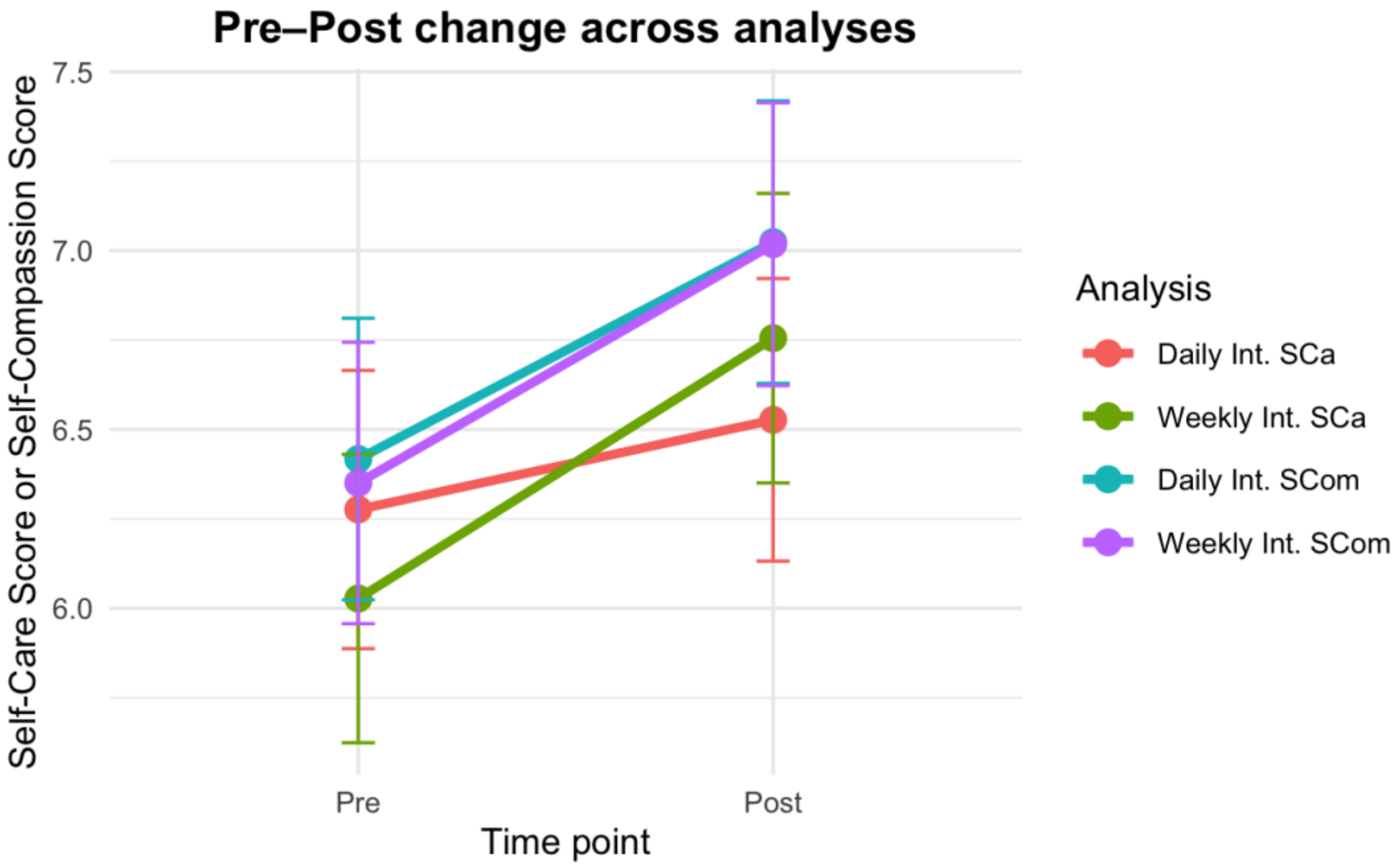

Figure SM 8. Estimated marginal means (EMMs) pre–post changes (±95% CI) in state self-care (SCa) and state self-compassion (SCom) across daily and weekly interventions.
Note. Values represent estimated marginal means with 95% confidence intervals for SCa and SCom in the daily and weekly intervention formats. Sample sizes varied across analyses (n = 59–66) depending on intervention type and outcome. Pre and post refer to state assessments immediately before and after the respective daily or weekly training intervention.

Table SM 8. Pre–Post Improvements in State Self-Compassion (SCom) and State Self-Care (SCa) During Daily and Weekly Interventions (according to H13 and H14)

Outcome	Intervention Type	*b*	95 % CI	*t*	*df*	*p*
State self-compassion	Weekly self-compasssion intervention	0.69	[0.40, 0.97]	4.67	150	**< .001**
State self-compassion	Daily self-compassion intervention	0.59	[0.35, 0.83]	4.82	150	**< .001**
State self-care	Weekly self-care intervention	0.81	[0.40, 1.22]	3.87	137	**< .001**
State self-care	Daily self-care intervention	0.20	[−0.13, 0.53]	1.18	154	.240

Note. Positive estimates (*b*) indicate higher post-intervention scores, reflecting improvement in state self-compassion and state self-care. Sample sizes ranged from 59 to 66 participants, with 195 to 216 observations contributing to the respective models due to repeated assessments within participants. Degrees of freedom vary accordingly.

9. Analyses for H15 (dose-response analyses)

Main analysis: Linear dose–response models using robust regression

Table 9a. Standardized regression coefficients (β) predicting post-intervention outcomes from total intervention dose among active intervention users (number of weekly and daily interventions in resiLIR; baseline-adjusted robust regression model)

Outcome	*β*	*t*	*df*	*p*	95% CI	*n*
Resilience (SR score)	–0.10	–0.86	56	.395	[–0.34, 0.14]	59
Self-Care – Positive Experience	0.30	2.85	56	**.006**	[0.09, 0.51]	59
Self-Care – Pacing	0.23	1.54	56	.131	[–0.07, 0.52]	59
Self-Compassion	0.07	0.94	56	.350	[–0.08, 0.23]	59
Burnout – Emotional Exhaustion	0.02	0.28	56	.784	[–0.11, 0.15]	59
Burnout – Depersonalization	0.08	0.92	54	.364	[–0.09, 0.24]	57
Burnout – Personal Accomplishment	0.07	0.85	56	.398	[–0.09, 0.22]	59

Note. Standardized regression coefficients (β) were obtained from baseline-adjusted robust linear regression models predicting post-intervention outcome from the total intervention dose (daily + weekly interventions). Robust regression was used to reduce the influence of extreme and influential observations without excluding participants. Analyses included active intervention users with available data on intervention dose and the respective baseline and post-intervention outcome (n = 57–59, depending on the outcome). Higher scores indicate improvement for all outcomes except resilience (SR score), emotional exhaustion, and depersonalization. CI = confidence interval.

Sensitivity analysis: Linear dose–response models using ordinary least squares (OLS)

Table SM 9b. Standardized regression coefficients (β) predicting post-intervention outcomes from total intervention dose among active intervention users (baseline-adjusted OLS regression models)

Outcome	*β*	*t*	*df*	*p*	95% CI	*n*
Resilience (SR score)	–0.17	–1.42	56	.161	[–0.40, 0.07]	59
Self-Care – Positive Experience	0.34	3.45	56	**.001**	[0.14, 0.54]	59
Self-Care – Pacing	0.27	2.27	56	**.027**	[0.03, 0.51]	59
Self-Compassion	0.11	1.23	56	.224	[–0.07, 0.29]	59
Burnout – Emotional Exhaustion	0.00	0.03	56	.973	[–0.16, 0.17]	59
Burnout – Depersonalization	0.22	2.40	54	**.020**	[0.04, 0.40]	57
Burnout – Personal Accomplishment	0.07	0.81	56	.424	[–0.10, 0.23]	59

Note. Standardized regression coefficients (β) were obtained from baseline-adjusted ordinary least squares (OLS) regression models predicting post-intervention outcomes from total intervention dose (number of completed daily and weekly interventions). Analyses included active intervention users with available data on intervention dose and the respective baseline and post-intervention outcome (n = 57–59, depending on the outcome). Higher scores indicate improvement for all outcomes except resilience (SR score), emotional exhaustion, and depersonalization. CI = confidence interval.

Exploratory analysis: Logarithmic dose-response models using robust regression

Table SM 9c. Standardized regression coefficients (β) predicting post-intervention outcomes from log-transformed total intervention dose among active intervention users (baseline-adjusted robust regression models)

Outcome	*β*	*t*	*df*	*p*	95% CI	*n*
Resilience (SR score)	–0.11	–0.70	59	.485	[–0.43, 0.20]	59
Self-Care – Positive Experience	0.30	2.07	59	**.043**	[0.01, 0.59]	59
Self-Care – Pacing	0.35	2.06	59	**.044**	[0.01, 0.69]	59
Self-Compassion	0.09	1.25	59	.215	[–0.05, 0.22]	59
Burnout – Emotional Exhaustion	0.01	0.10	59	.919	[–0.09, 0.10]	59
Burnout – Depersonalization	0.05	0.24	56	.815	[–0.37, 0.46]	57
Burnout – Personal Accomplishment	0.12	1.54	59	.129	[–0.04, 0.28]	59

Note. Standardized regression coefficients (β) were obtained from robust logarithmic regression models (lmrob, robustbase package) predicting post-intervention outcomes (t2) from the log-transformed total intervention dose (daily + weekly interventions), while controlling for baseline (t0) levels of the respective outcome. Robust regression was used to reduce the influence of extreme and influential observations without excluding participants. Analyses included active intervention users with available data on intervention dose and the respective baseline and post-intervention outcome (n = 57–59, depending on the outcome). Higher scores indicate improvement for all outcomes except resilience (SR score), emotional exhaustion, and depersonalization. CI = confidence interval.

10. Sensitivity analyses for H1-12 and H15

Per protocol analyses with participants completing minimum 14 interventions

Table SM 10a. Results of PP sensitivity analysis (with participants who completed at least 14 interventions) of hypotheses H1 to H11: Overall group x time effects

**Hypothesis: Outcome**	**b**	**SE**	**95% CI**	**df**	**t**	***p***	**Interpretation: Effect for intervention group**
H1: Resilience (SR Score)	–0.31	0.12	[–0.56, –0.07]	327.82	–2.53	**.012**	Significant favourable effect (lower SR Score)
H2: Resilience (BRS)	0.18	0.09	[–0.00, 0.37]	295.61	1.94	.053	Close-to-significant favourable trend
H3a: Self-Care – Positive Experience	0.33	0.11	[0.11, 0.54]	325.36	2.99	**.003**	Significant favourable effect
H3b: Self-Care - Pacing	0.57	0.12	[0.33, 0.80]	320.32	4.77	**<.001**	Significant favourable effect
H4: Self-Compassion	0.25	0.07	[0.12, 0.38]	268.10	3.74	**<.001**	Significant favourable effect
H5: Mindfulness	0.16	0.11	[–0.05, 0.38]	301.54	1.50	.135	No effect
H6a: Perceived Stress – subfactor helplessness	–0.14	0.12	[–0.39, 0.10]	323.14	–1.15	.249	No effect
H6b: Perceived Stress – subfactor self-efficacy	0.37	0.10	[0.18, 0.56]	323.90	3.89	**<.001**	Significant favourable effect
H7: Mental Distress (GHQ)	–3.67	0.80	[–5.24, –2.10]	326.25	–4.57	**<.001**	Significant favourable effect
H8: Depression	–1.38	0.57	[–2.49, –0.27]	319.36	–2.43	**.016**	Significant favourable effect
H9: Anxiety	–1.51	0.53	[–2.55, –0.47]	327.02	–2.85	**.005**	Significant favourable effect
H10a: Burnout – subfactor Emotional Exhaustion	–0.16	0.13	[–0.42, 0.10]	264.99	–1.20	.230	No effect
H10b: Burnout – subfactor Depersonalisation	–0.21	0.16	[–0.53, 0.12]	298.76	–1.26	.210	No effect
H10c: Burnout – subfactor Personal Accomplishment	0.15	0.10	[–0.05, 0.35]	302.87	1.50	.134	No effect
H11: Work-Life-Balance	0.06	0.13	[–0.19, 0.31]	308.86	0.48	.635	No effect

Note. Negative coefficients (b) indicate improvements (reductions) for negatively oriented outcomes (i.e., resilience (SR Score), helplessness, mental distress, depression, anxiety, emotional exhaustion, and depersonalisation). Positive coefficients indicate improvement for positively oriented outcomes (i.e., resilience (BRS), self-care, self-compassion, mindfulness, self-efficacy, personal accomplishment, and work-life-balance).

Table SM 10b. Results of PP sensitivity analysis (with participants who completed at least 14 interventions) for hypotheses H1 to H11: Specific group x time effects for post-intervention and first follow-up (3 months after completion of intervention)

Hypothesis: Outcome	Week	b	SE	95% CI	Df	t	*p*
H1: Resilience (SR Score)	9	–0.39	0.15	[–0.69, –0.10]	310.21	–2.64	**.009**
	21	–0.21	0.16	[–0.52, 0.11]	305.42	–1.30	.196
H2: Resilience (BRS)	9	0.17	0.11	[–0.04, 0.38]	272.09	1.55	.122
	21	0.19	0.12	[–0.04, 0.42]	267.49	1.63	.104
H3a: Self-Care – Positive Experience	9	0.35	0.13	[0.09, 0.60]	300.73	2.66	**.008**
	21	0.30	0.14	[0.02, 0.57]	295.41	2.13	**.034**
H3b: Self-Care – Pacing	9	0.69	0.14	[0.42, 0.96]	292.98	4.96	**<.001**
	21	0.43	0.15	[0.14, 0.72]	287.72	2.87	**.004**
H4: Self-Compassion	9	0.19	0.08	[0.04, 0.34]	251.63	2.47	**.014**
	21	0.33	0.08	[0.16, 0.49]	248.40	3.94	**<.001**
H5: Mindfulness	9	0.07	0.13	[–0.18, 0.32]	275.94	0.53	.595
	21	0.30	0.14	[0.03, 0.56]	271.06	2.19	**.029**
H6a: Perceived Stress – subfactor helplessness	9	–0.27	0.15	[–0.57, 0.03]	316.45	–1.79	.075
	21	0.01	0.16	[–0.31, 0.33]	312.46	0.04	.966
H6a: Perceived Stress – subfactor self-efficacy	9	0.44	0.11	[0.22, 0.66]	298.46	3.90	**<.001**
	21	0.28	0.12	[0.04, 0.52]	293.20	2.29	**.023**
H7: Mental distress (GHQ)	9	–4.13	0.98	[–6.05, –2.22]	314.07	–4.24	**<.001**
	21	–3.09	1.05	[–5.16, –1.03]	309.75	–2.93	**.004**
H8: Depression (PHQ)	9	–1.50	0.67	[–2.82, –0.18]	292.90	–2.22	**.027**
	21	–1.25	0.72	[–2.67, 0.17]	287.49	–1.72	.086
H9: Anxiety (GAD)	9	–1.81	0.64	[–3.06, –0.57]	303.78	–2.85	**.005**
	21	–1.13	0.69	[–2.47, 0.21]	298.54	–1.65	.100
H10a: Burnout – subfactor Emotional Exhaustion	9	–0.06	0.15	[–0.36, 0.24]	249.18	–0.37	.709
	21	–0.29	0.16	[–0.61, 0.03]	246.03	–1.77	.079
H10b: Burnout – subfactor Depersonalisation	9	–0.04	0.19	[–0.41, 0.34]	273.21	–0.20	.840
	21	–0.42	0.21	[–0.82, –0.01]	268.09	–2.03	.043
H10c: Burnout – subfactor Personal Accomplishment	9	0.11	0.12	[–0.12, 0.35]	278.59	0.96	.339
	21	0.20	0.13	[–0.05, 0.45]	273.75	1.58	.115
H11: Work-Life-Balance	9	–0.08	0.15	[–0.38, 0.21]	282.12	–0.55	.585
	21	0.24	0.16	[–0.08, 0.56]	277.00	1.50	.136

Note. Week 9 = post-intervention; week 21 = follow-up (3 months after completion of the intervention). Negative coefficients (b) indicate improvements (reductions) for negatively oriented outcomes (i.e., resilience (SR Score), helplessness, mental distress, depression, anxiety, emotional exhaustion, and depersonalisation). Positive coefficients indicate improvement for positively oriented outcomes (i.e., resilience (BRS), self-care, self-compassion, mindfulness, self-efficacy, personal accomplishment, and work-life-balance).

11. Moderator and dropout analyses

Additional analyses for moderation in ITT dataset:

Moderation analyses

Primary outcome: SR Score

Across all moderation models – Attitudes toward online interventions (APOI), confidence in online interventions (APOI subscale), satisfaction with the intervention (CSQ), satisfaction with the intervention, technical difficulties, age and sex – none of the effects reached significance (all $ps \geq .191$).

Secondary outcomes: Self-Care, SC, GHQ

Within the intervention group: Feedback and technical difficulties were unrelated to any outcome (all $ps \geq .23$). Only client satisfaction (CSQ) was significantly associated with lower GHQ scores ($p = .012$), but not with self-care or self-compassion (all $ps \geq .19$).
Between intervention and control group: For self-care and GHQ, none of the variables (APOI, age, sex) significantly moderated the intervention effect (all interaction $ps \geq .34$).

Dropout analyses

Completers and dropouts did not differ on any baseline characteristics, including SR Score, stress (on Kessler 10 Distress Scale), attitudes toward online interventions (APOI), age, or sex (all $ps \geq .139$).

12. Exploratory within-group analyses for self-compassion, mindfulness and resilience (BRS)

Table SM 12a. Linear Mixed Model Results for Self-Compassion (only Intervention Group; PP analysis)

**Assessment (weeks after baseline)**	***b***	**95 % CI**	***p***	**Interpretation**
Intermediate (4)	0.19	[0.07, 0.31]	.003	Significant increase in self-compassion
Post-intervention (9)	0.26	[0.13, 0.39]	< .001	Highly significant increase in self-compassion
3-months Follow-Up (21)	0.39	[0.25, 0.52]	< .001	Highly significant increase in self-compassion
6-months Follow-Up (33)	0.37	[0.23, 0.50]	< .001	Highly significant increase in self-compassion
1-year Follow-Up (59)	0.33	[0.19, 0.47]	< .001	Sustained highly significant increase in self-compassion

Note. n = 48

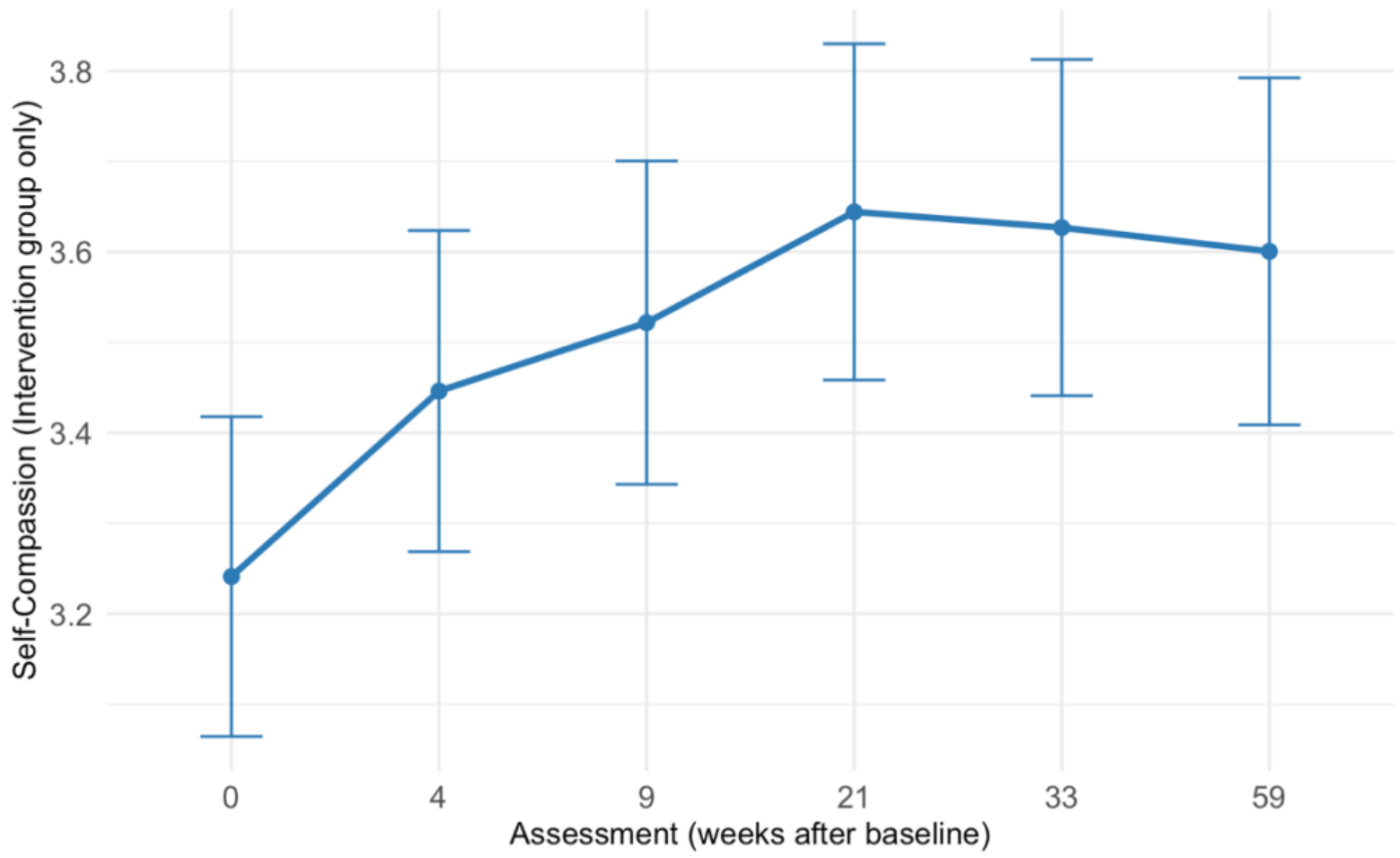

Figure SM 12a. Development of Self-compassion score (SCS) in Intervention Group over assessments (PP analysis).
Note. Values represent model-based estimated marginal means (EMMs) from a linear mixed-effects model fitted to the intervention group only. Error bars indicate 95% confidence intervals. *n* = 48.

Table SM 12b. Linear Mixed Model Results for Mindfulness (only Intervention Group; PP analysis)

**Assessment (weeks after baseline)**	***b***	**95 % CI**	***p***	**Interpretation**
Intermediate (4)	–0.03	[–0.19, 0.14]	.746	No change
Post-intervention (9)	–0.01	[–0.18, 0.16]	.930	No change
3-months Follow-Up (21)	0.24	[0.06, 0.42]	.009	Significant increase in mindfulness
6-months Follow-Up (33)	0.07	[–0.11, 0.25]	.458	No change
1-year Follow-Up (59)	0.18	[–0.01, 0.37]	.059	Close-to-significant favourable trend

Note. n = 48

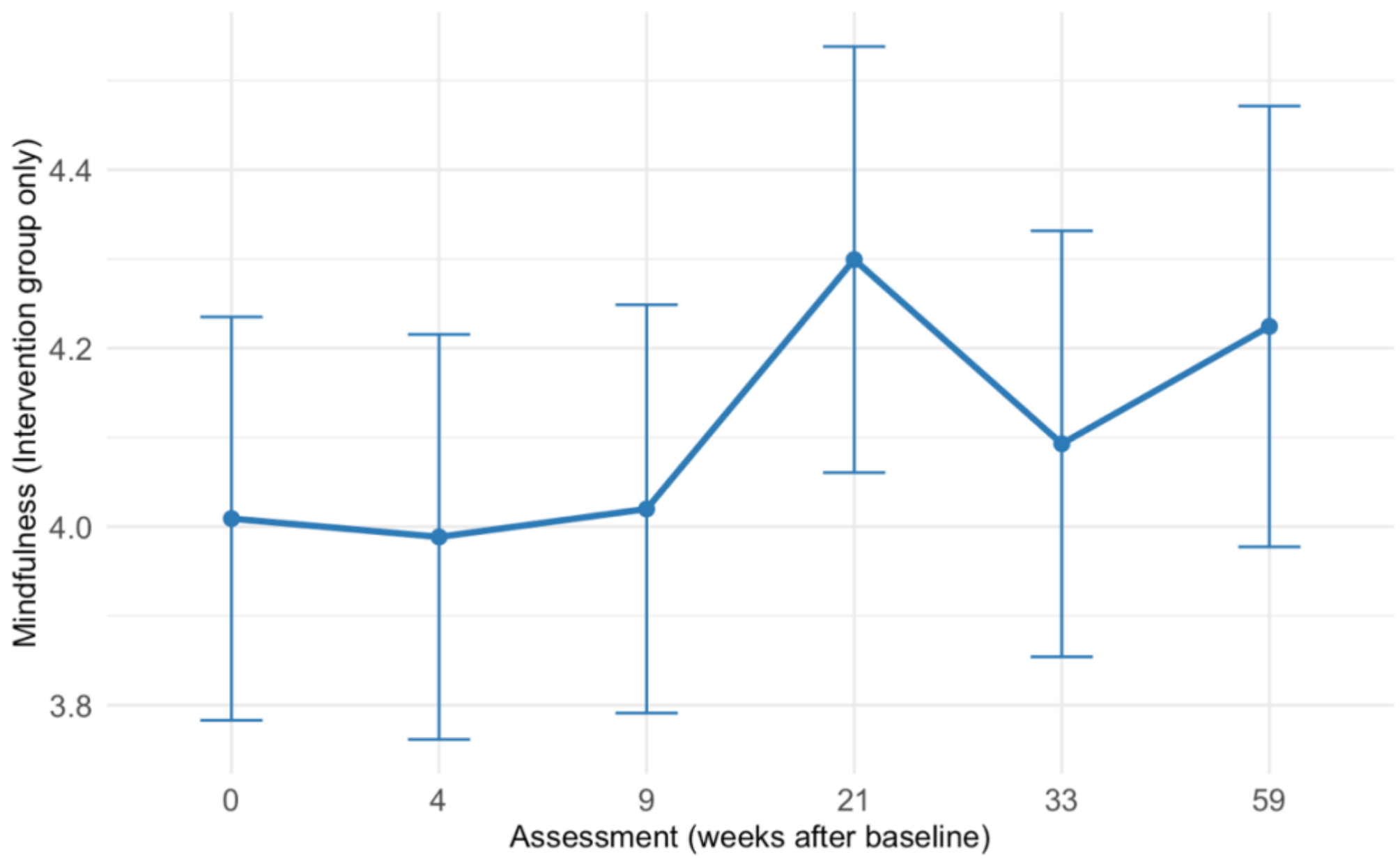

Figure SM 12b. Development of Mindfulness score (MAAS) in Intervention Group over assessments (PP analysis).
Note. Values represent model-based estimated marginal means (EMMs) from a linear mixed-effects model fitted to the intervention group only. Error bars indicate 95% confidence intervals. *n* = 48.

Table SM 12c. Linear Mixed Model Results for Resilience measured with the Brief Resilience Scale (BRS; only Intervention Group; PP analysis)

**Assessment (weeks after baseline)**	***b***	**95 % CI**	***p***	**Interpretation**
Intermediate (4)	0.14	[–0.02, 0.29]	.085	No significant change
Post-intervention (9)	0.32	[0.17, 0.48]	< .001	Significant increase in resilience
3-months Follow-Up (21)	0.30	[0.13, 0.46]	< .001	Significant increase in resilience
6-months Follow-Up (33)	0.31	[0.14, 0.47]	< .001	Significant increase in resilience
1-year Follow-Up (59)	0.34	[0.17, 0.52]	< .001	Significant increase in resilience

Note. n = 48

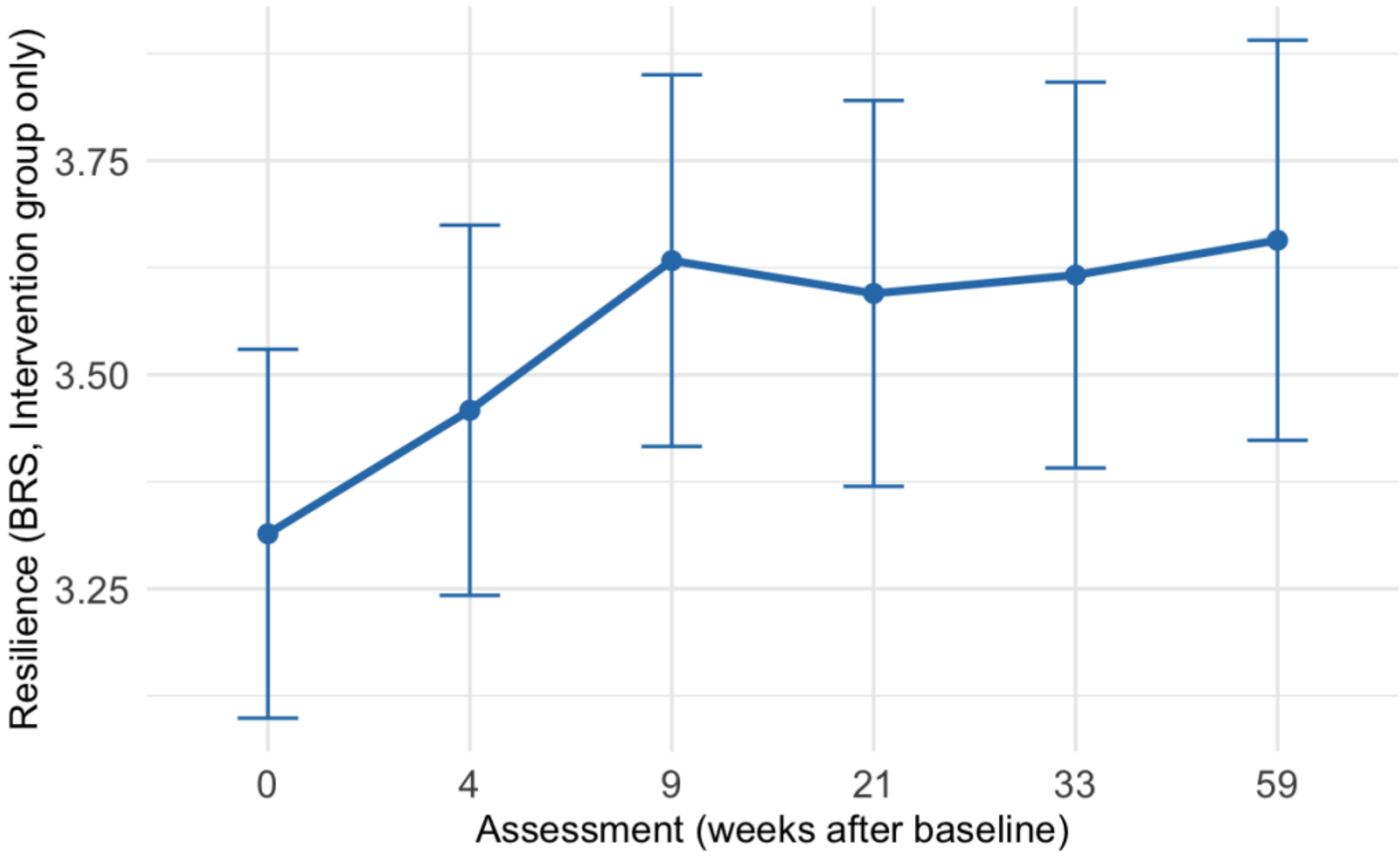

Figure SM 12c. Development of Brief Resilience Scale (BRS) Score in Intervention Group over assessments (PP analysis).
Note. Values represent model-based estimated marginal means (EMMs) from a linear mixed-effects model fitted to the intervention group only. Error bars indicate 95% confidence intervals. *n* = 48.

13. Assessment of potential negative effects of the intervention (INEP-ON)

Description of instrument: INEP-ON
Negative and positive effects of the intervention were assessed using the *Inventory of Negative Effects of Online Interventions* (INEP-ON; Ladwig et al., 2014). The instrument comprises 23 items. The first 11 items are bipolar and assess both positive and negative changes following the intervention (e.g., feeling better/worse, increased/decreased loneliness). Responses were given on a 7-point scale ranging from −3 (maximal negative change) to +3 (maximal positive change), with 0 indicating no change/neutral. Mean scores were calculated, with higher positive values reflecting more positive perceived effects and negative values reflecting more negative perceived effects.
Items 12–23 assess exclusively negative effects (e.g., financial worries, prolonged periods of feeling bad, data protection concerns) and were rated on a 4-point scale ranging from 0 (no negative effect) to 3 (severe negative effect).
For Items 1–21, participants additionally indicated whether they attributed the perceived changes to the intervention or to their life circumstances.

Negative effects of online interventions.
Across the 11 bipolar items assessing positive and negative intervention effects, negative changes were reported by a minority of participants (n = 14, 20%), whereas the vast majority (n = 65, 94%) reported at least one positive effect. Reported negative changes were infrequent at the item level and each was endorsed by only a small number of participants. Importantly, five participants (7%) attributed at least one negative effect within these items to the intervention.

Across the additional 12 items assessing exclusively negative effects, 53 participants reported at least one mild to severe negative experience. However, these were predominantly mild, while moderate and severe effects were rare. Twelve participants (18%) attributed at least one negative effect within these items to the intervention, corresponding to 17 intervention-attributed negative ratings in total. Across all 23 INEP-ON items, 10 participants (approximately 15%) attributed any negative effect to the intervention.
Details in Figures SM 9a and SM 9b, and Table SM 9.

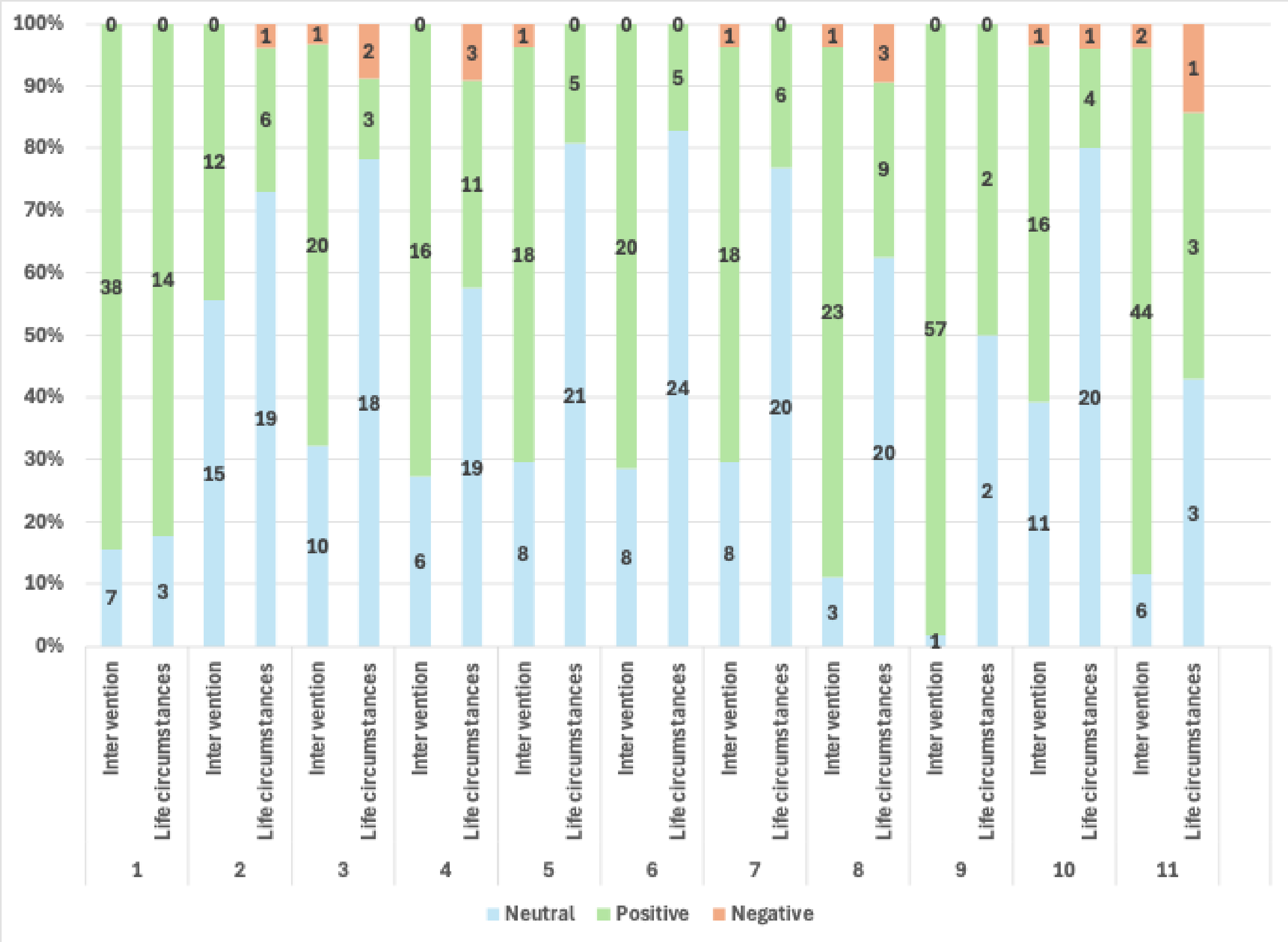

Figure SM 9a. INEP-ON items 1–11: Positive, neutral, and negative effects by attribution (numbers in bars refer to participants per category).

Note. Each item is shown separately by attribution category, i.e., if participants attributed the effect to the intervention or their life circumstances. Numbers below the x-axis indicate INEP-ON item numbers. The items assess the following potential effects of the online intervention: 1 = Feeling better/worse; 2 = Easier/harder to trust; 3 = Suffer less/more; 4 = Fewer/more conflicts in relationship; 5 = Improved/worsened relationship with family; 6 = Improved/worsened relationship with friends; 7 = Decreased/increased loneliness; 8 = Increased/decreased hobbies and social contacts; 9 = Helpful/harmful ways of thinking learned; 10 = Increased/decreased motivation to start psychotherapy; 11 = Supportive/disturbing guide.

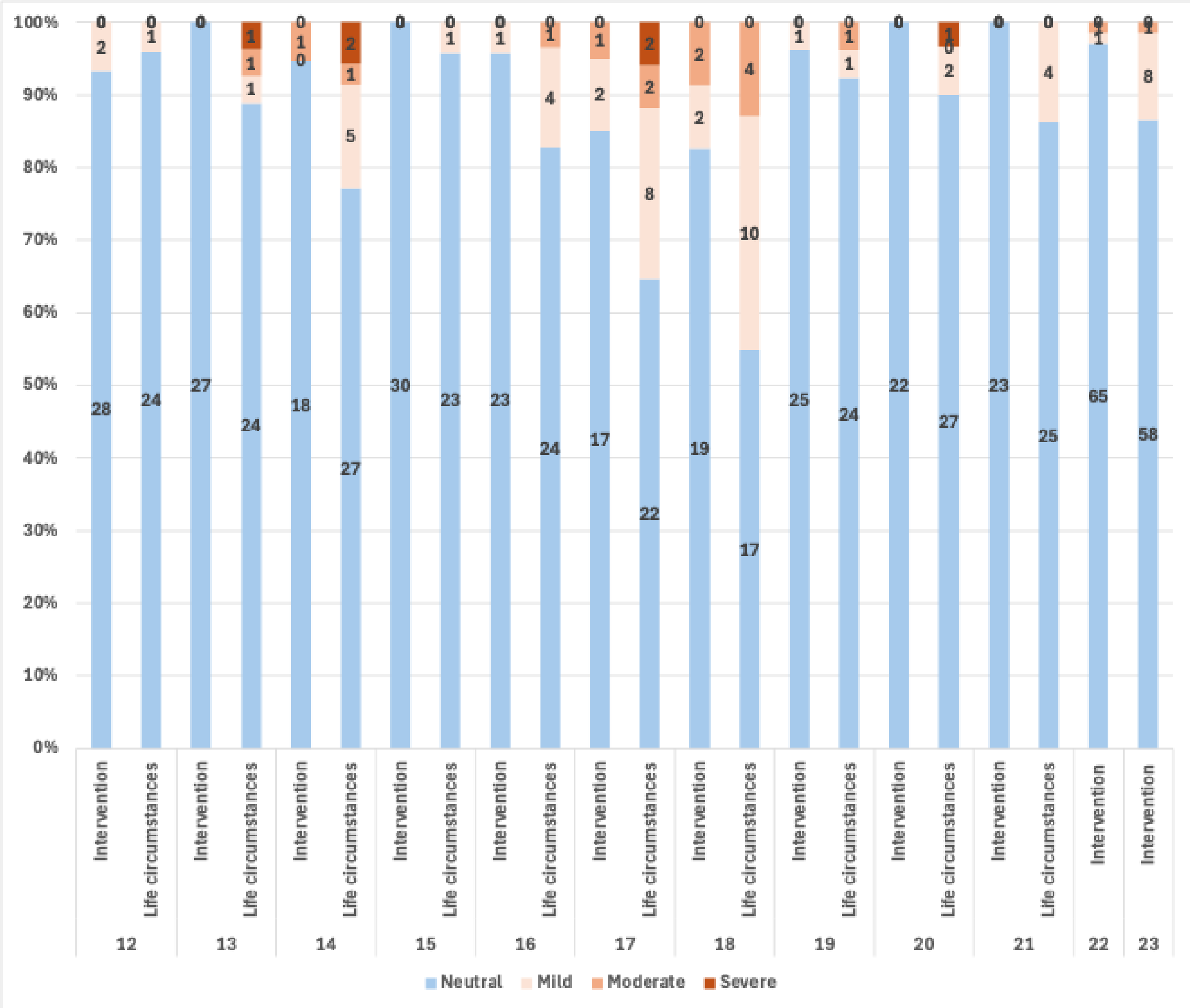

Figure SM 9b. INEP-ON items 12–23: Negative effects (neutral, mild, moderate, severe) by attribution.

Note. Each item is shown separately by attribution category, i.e., if participants attributed the effect to the intervention or their life circumstances. Items 22 and 23 refer exclusively to intervention-related experiences. Numbers below the x-axis indicate INEP-ON item numbers. The items assess the following potential negative effects of the online intervention: 12 = Disclosure concerns (fear that others might find out about intervention use); 13 = Insurance problems or concerns; 14 = Financial worries; 15 = Dependency on the intervention; 16 = Difficulty making important decisions; 17 = Relationship problems; 18 = Prolonged periods of feeling bad; 19 = Changed for the worse as a person; 20 = First-time suicidal thoughts or intentions; 21 = Increased alcohol/drug consumption; 22 = Hurtful intervention content; 23 = Data protection concerns.

Table SM 9. Descriptive statistics for Inventory of Negative Effects of Online-Interventions (INEP-ON) within the IG at t2

	Overall			Attributed to intervention			Attributed to life circumstances			No attribution
**Assessment (weeks after baseline)**	***N***	***M***	***SD***	***n (%)***	***M***	***SD***	***n (%)***	***M***	***SD***	***n (%)***
1: Feeling better/worse	69	1.28	0.86	45 (65.2%)	1.47	0.87	17 (24.6%)	1.12	0.70	7 (10.1%)
2: Easier/harder to trust	69	0.36	0.69	27 (39.1%)	0.59	0.80	26 (37.7%)	0.27	0.67	16 (23.2%)
3: Suffer less/more	69	0.43	0.79	31 (44.9%)	0.87	0.88	23 (33.3%)	0.09	0.60	15 (21.7%)
4: Fewer/more conflicts in relationship	68	0.59	0.95	22 (32.4%)	0.95	0.72	33 (48.5%)	0.48	1.12	13 (19.1%)
5: Improved/worsened relationship with family	68	0.46	0.74	27 (39.7%)	0.85	0.82	26 (38.2%)	0.23	0.51	15 (22.1%)
6: Improved/worsened relationship with friends	68	0.53	0.74	28 (41.2%)	0.93	0.77	29 (42.6%)	0.21	0.49	11 (16.2%)
7: Decreased/increased loneliness	68	0.56	0.87	27 (39.7%)	1.07	1.04	26 (38.2%)	0.27	0.53	15 (22.1%)
8: Increased/decreased hobbies and social contacts	68	0.65	1.00	27 (39.7%)	1.19	1.04	32 (47.1%)	0.28	0.89	9 (13.2%)
9: Helpful/harmful ways of thinking learned	68	1.84	0.91	58 (85.3%)	2.02	0.81	4 (5.9%)	0.50	0.58	6 (8.8%)
10: Increased/decreased motivation to start psychotherapy	68	0.44	0.82	28 (41.2%)	0.75	0.89	25 (36.8%)	0.24	0.78	15 (22.1%)
11: Supportive/disturbing guide	67	1.33	1.05	52 (77.6%)	1.52	1.04	7 (10.4%)	0.29	0.76	8 (11.9%)
12: Disclosure concerns	67	0.06	0.24	30 (44.8%)	0.07	0.26	25 (37.3%)	0.04	0.20	12 (17.9%)
13: Insurance problems/concerns	67	0.09	0.45	27 (40.3%)	0.00	0.00	27 (40.3%)	0.12	0.55	13 (19.4%)
14: Financial worries	67	0.22	0.65	19 (28.4%)	0.05	0.22	35 (52.2%)	0.31	0.74	13 (19.4%)
15: Dependency on intervention	67	0.01	0.12	30 (44.8%)	0.00	0.00	24 (35.8%)	0.04	0.20	13 (19.4%)
16: Difficulty making decisions	67	0.10	0.35	24 (35.8%)	0.04	0.20	29 (43.3%)	0.17	0.47	14 (20.9%)
17: Relationship problems	67	0.36	0.73	20 (29.9%)	0.15	0.37	34 (50.7%)	0.50	0.87	13 (19.4%)
18: Prolonged periods of feeling bad	67	0.39	0.65	23 (34.3%)	0.22	0.52	31 (46.3%)	0.52	0.74	13 (19.4%)
19: Changed for the worse as a person	67	0.06	0.30	26 (38.8%)	0.04	0.20	26 (38.8%)	0.08	0.39	15 (22.4%)

Assessment (weeks after baseline)	Overall			Attributed to intervention			Attributed to life circumstances			No attribution
	N	*M*	*SD*	*n (%)*	*M*	*SD*	*n (%)*	*M*	*SD*	*n (%)*
20: First-time suicidal thoughts	67	0.07	0.40	22 (32.8%)	0.00	0.00	30 (44.8%)	0.10	0.56	15 (22.4%)
21: Increased alcohol/drug consumption	67	0.06	0.24	23 (34.3%)	0.00	0.00	29 (43.3%)	0.08	0.28	15 (22.4%)
22: Hurtful intervention content	67	0.04	0.27	—	—	—	—	—	—	—
23: Data protection concerns	67	0.15	0.40	—	—	—	—	—	—	—

Note. Items 1–11 are bipolar and assess positive as well as negative changes. Responses were coded from −3 to +3, with negative values indicating negative changes, 0 indicating no change, and positive values indicating positive changes. Thus, higher positive mean values reflect more positive perceived effects, whereas lower (negative) mean values reflect more negative perceived effects.
Items 12–23 assess exclusively negative effects and were coded from 0 to 3 (0 = no negative effect, 1 = mild, 2 = moderate, 3 = severe). Accordingly, higher mean values indicate more severe negative effects.
For Items 1–21, participants additionally indicated whether perceived changes were attributable to the intervention or to life circumstances. If no attribution was provided, responses were categorized as “no attribution. Items 22 and 23 are inherently intervention-related.
Percentages refer to the proportion of participants with valid responses for the respective item.

14. Sensitivity analysis of H1 using an SR Score based on an HCP-only normative regression

Rationale. The original SR Score was calculated using a normative regression estimated from the HCP sample supplemented with baseline data from two sister trials. For the sensitivity analysis, the normative regression was re-estimated using baseline data from the HCP sample only.
Result. Effect estimates were highly comparable between the original and HCP-only SR Score calculations, and the substantive conclusions remained unchanged in both PP and ITT analyses.

PP analysis: Overall group x time effects

**Hypothesis: Outcome**	**b**	**SE**	**95% CI**	**df**	**t**	***p***
H1: Resilience (SR Score)	–0.29	0.11	[–0.51, –0.07]	361	–2.63	**.009**
H1: Resilience (SR Score) *(SR Score based on HCP sample only.)*	–0.29	0.11	[–0.51, –0.07]	361	–2.57	**.010**

PP analysis: Specific group x time effects for post-intervention and first follow-up (3 months after completion of intervention)

**Hypothesis: Outcome**	**Week**	**b**	**SE**	**95% CI**	**Df**	**t**	***p***
H1: Resilience (SR Score)	9	–0.34	0.13	[–0.60, –0.08]	339	–2.55	**.011**
	21	–0.22	0.14	[–0.51, 0.06]	333	–1.55	.121
H1: Resilience (SR Score) *(SR Score based on HCP sample only.)*	9	–0.34	0.13	[–0.60, –0.07]	339	–2.50	**.013**
	21	–0.22	0.15	[–0.50, 0.07]	334	–1.50	.134

Note. Week 9 = post-intervention; week 21 = follow-up (3 months after completion of the intervention).

ITT analysis: Overall group x time effects

**Hypothesis: Outcome**	**b**	**SE**	**95% CI**	**df**	**t**	***p***
H1: Resilience (SR Score)	-0.10	0.09	[-0.28, 0.08]	188	-1.13	.261
H1: Resilience (SR Score) *(SR Score based on HCP sample only.)*	–0.11	0.10	[–0.30, 0.09]	128	–1.08	.280

ITT analysis: Specific group x time effects for post-intervention and first follow-up (3 months after completion of intervention)

**Hypothesis: Outcome**	**Week**	**b**	**SE**	**95% CI**	**Df**	**t**	***p***
H1: Resilience (SR Score)	9	-0.16	0.12	[-0.39, 0.07]	137	-1.41	.160
	21	-0.04	0.11	[-0.26, 0.18]	191	-0.39	.701
H1: Resilience (SR Score) *(SR Score based on HCP sample only.)*	9	–0.16	0.12	[–0.39, 0.08]	127	–1.34	.184
	21	–0.05	0.12	[–0.29, 0.19]	113	–0.43	.668

Note. Week 9 = post-intervention; week 21 = follow-up (3 months after completion of the intervention).

15. References in Supplementary Material

Büssing, A., & Perrar, K. M. (1992). Die Messung von Burnout. Untersuchung einer deutschen Fassung des Malach Burnout Inventars. *Diagnostica*, *38*(4), 328–353.

Chmitorz, A., Kurth, K., Mey, L. K., Wenzel, M., Lieb, K., Tüscher, O., Kubiak, T., & Kalisch, R. (2020). Assessment of Microstressors in Adults: Questionnaire Development and Ecological Validation of the Mainz Inventory of Microstressors. *JMIR Mental Health*, *7*(2), e14566. https://doi.org/10.2196/14566

Chmitorz, A., Wenzel, M., Stieglitz, R.-D., Kunzler, A., Bagusat, C., Helmreich, I., Gerlicher, A., Kampa, M., Kubiak, T., Kalisch, R., Lieb, K., & Tüscher, O. (2018). Population-based validation of a German version of the Brief Resilience Scale. *PLOS ONE*, *13*(2), e0192761. https://doi.org/10.1371/journal.pone.0192761

Eisinga, R., Grotenhuis, M. T., & Pelzer, B. (2013). The reliability of a two-item scale: Pearson, Cronbach, or Spearman-Brown? *International Journal of Public Health*, *58*(4), 637–642. https://doi.org/10.1007/s00038-012-0416-3

Harfst, T., Ghods, C., Mösko, M., & Schulz, H. (2009). Erfassung von positivem Verhalten und Erleben bei Patienten mit psychischen und psychosomatischen Erkrankungen in der Rehabilitation – der Hamburger Selbstfürsorgefragebogen (HSF). *Die Rehabilitation*, *48*(05), 277–282. https://doi.org/10.1055/s-0029-1239546

Höfling, V., Moosbrugger, H., Schermelleh-Engel, K., & Heidenreich, T. (2011). Mindfulness or Mindlessness?: A Modified Version of the Mindful Attention and Awareness Scale (MAAS). *European Journal of Psychological Assessment*, *27*(1), 59–64. https://doi.org/10.1027/1015-5759/a000045

Hupfeld, J., & Ruffieux, N. (2011). Validierung einer deutschen Version der Self-Compassion Scale (SCS-D). *Zeitschrift für Klinische Psychologie und Psychotherapie*, *40*(2), 115–123. https://doi.org/10.1026/1616-3443/a000088

Kroenke, K., Spitzer, R. L., & Williams, J. B. W. (2001). The PHQ-9: Validity of a brief depression severity measure. *Journal of General Internal Medicine*, *16*(9), 606–613. https://doi.org/10.1046/j.1525-1497.2001.016009606.x

Ladwig, I., Rief, W., & Nestoriuc, Y. (2014). Welche Risiken und Nebenwirkungen hat Psychotherapie? - Entwicklung des Inventars zur Erfassung Negativer Effekte von Psychotherapie (INEP). *Verhaltenstherapie*, *24*(4), 252–263. https://doi.org/10.1159/000367928

Löwe, B., Spitzer, R. L., Zipfel, S., & Herzog, W. (2002). *Gesundheitsfragebogen für Patienten (PHQ-D). Manual und Testunterlagen.* (2. Auflage). Pfizer.

Schäfer, S. K., Von Boros, L., Göritz, A. S., Baumann, S., Wessa, M., Tüscher, O., Lieb, K., & Möhring, A. (2023). The Perceived Stress Scale 2&2: A two-factorial German short version of the Perceived Stress Scale. *Frontiers in Psychiatry*, *14*, 1195986. https://doi.org/10.3389/fpsyt.2023.1195986

Schrnitz, N., Kruse, J., & Tress, W. (1999). Psychometric properties of the General Health Questionnaire (GHQ-12) in a German primary care sample. *Acta Psychiatrica Scandinavica*, *100*(6), 462–468. https://doi.org/10.1111/j.1600-0447.1999.tb10898.x

Spitzer, R. L., Kroenke, K., Williams, J. B. W., & Löwe, B. (2006). A Brief Measure for Assessing Generalized Anxiety Disorder: The GAD-7. *Archives of Internal Medicine*, *166*(10), 1092. https://doi.org/10.1001/archinte.166.10.1092

Syrek, C., Bauer-Emmel, C., Antoni, C., & Klusemann, J. (2011). Entwicklung und Validierung der Trierer Kurzskala zur Messung von Work-Life Balance (TKS-WLB). *Diagnostica*, *57*(3), 134–145. https://doi.org/10.1026/0012-1924/a000044
